# NLRP10 Enhances CD4^+^ T-Cell-Mediated IFNγ Response *via* Regulation of Dendritic Cell-Derived IL-12 Release

**DOI:** 10.3389/fimmu.2017.01462

**Published:** 2017-11-02

**Authors:** Maurizio Vacca, Julia Böhme, Lia Paola Zambetti, Hanif Javanmard Khameneh, Bhairav S. Paleja, Federica Laudisi, Adrian W. S. Ho, Kurt Neo, Keith Weng Kit Leong, Mardiana Marzuki, Bernett Lee, Michael Poidinger, Laura Santambrogio, Liana Tsenova, Francesca Zolezzi, Gennaro De Libero, Amit Singhal, Alessandra Mortellaro

**Affiliations:** ^1^Singapore Immunology Network (SIgN), Agency for Science, Technology and Research (A*STAR), Singapore, Singapore; ^2^Department of Microbiology and Immunology, Yong Loo Lin School of Medicine, National University of Singapore, Singapore, Singapore; ^3^Department of Pathology, Albert Einstein College of Medicine, New York, NY, United States; ^4^Department of Biological Sciences, New York City College of Technology, Brooklyn, NY, United States; ^5^Department of Biomedicine, University of Basel and University Hospital Basel, Basel, Switzerland; ^6^Lee Kong Chian School of Medicine, Nanyang Technological University, Singapore, Singapore

**Keywords:** NLRP10, dendritic cells, CpG DNA, toll-like receptor 9, IL-12, T helper 1, IFNγ, *Mycobacterium tuberculosis*

## Abstract

NLRP10 is a nucleotide-binding oligomerization domain-like receptor that functions as an intracellular pattern recognition receptor for microbial products. Here, we generated a *Nlrp10^−/−^* mouse to delineate the role of NLRP10 in the host immune response and found that *Nlrp10^−/−^* dendritic cells (DCs) elicited sub-optimal IFNγ production by antigen-specific CD4^+^ T cells compared to wild-type (WT) DCs. In response to T-cell encounter, CD40 ligation or Toll-like receptor 9 stimulation, *Nlrp10^−/−^* DCs produced low levels of IL-12, due to a substantial decrease in NF-κB activation. Defective IL-12 production was also evident *in vivo* and affected IFNγ production by CD4^+^ T cells. Upon *Mycobacterium tuberculosis* (*Mtb*) infection, *Nlrp10^−/−^* mice displayed diminished T helper 1-cell responses and increased bacterial growth compared to WT mice. These data indicate that NLRP10-mediated IL-12 production by DCs is critical for IFNγ induction in T cells and contributes to promote the host defense against *Mtb*.

## Introduction

Dendritic cells (DCs) sense infectious agents through germline-encoded pattern recognition receptors (PRRs) that recognize the molecular patterns expressed by micro-organisms and endogenous stimuli. PRR activation induces signaling cascades that initiate an inflammatory response elicited in part, by cytokine production. The membrane-bound and endosomal Toll-like receptors (TLRs) are PRRs that have been most extensively investigated (1), but recent research has discovered families of cytosolic PRRs, including nucleotide-binding oligomerization domain (NOD)-like receptors (NLRs) (2). Some of these sensors can form an inflammasome that regulates the activation of caspase-1 to drive IL-1β and IL-18 cytokine processing and release (3). The best-characterized inflammasome complex comprises the inflammasome sensor NLRP3, the adaptor ASC and the caspase-1 precursor. This inflammasome can be activated by environmental stimuli and a plethora of chemically and structurally different stimuli, including pathogen-associated molecular patterns (PAMPs) and danger-associated molecular patterns that are released during cellular damage [reviewed in Ref. (4)].

NLRP proteins are involved in pathogen recognition and inflammatory and immune responses to microbial infections. NLRP6 contributes to intestinal homeostasis by preventing pathogenic colitis associated with microbial dysbiosis and regulates intestinal mucosa tissue repair (5). NLRP12 has been linked to *Yersinia pestis* infection recognition (6), and exhibits an inflammasome-independent anti-inflammatory function that protects mice from experimental colitis by inhibiting the canonical and non-canonical NF-κB pathways (7). Despite such research efforts our knowledge as to how NLRs modulate the immune response is limited and many NLRs remain poorly characterized.

NLRP10 is an NLR that lacks the characteristic C-terminal leucine-rich (LRR) domain present in most members of this protein family (8). Over-expression studies in mice showed that human NLRP10 negatively regulated NF-κB and cell death and inhibited IL-1β release *in vivo* and *in vitro* (9). These NLRP10 knock-in mice showed resistance to lipopolysaccharide (LPS)-induced endotoxic shock, likely due to decreased inflammatory cytokine release (9). Furthermore, peritoneal macrophages from these mice released low levels of IL-1β in response to *Salmonella enterica* serovar *Typhimurium* infection or TLR7 stimulation (9). Despite such support for a role of NLRP10 in IL-1β processing and release, other studies have not provided evidence that NLRP10 contributes to inflammasome activation (10, 11). Consequently, an inflammasome-independent function of NLRP10 has been suggested, based on interactions between NLRP10, NOD1, and its signaling partners RIPK2, TAK1, and NEMO in human epithelial cells (12). More recently, NLRP10 has been implicated in immune response to *Leishmania major* (13).

A role for NLRP10 in adaptive immunity has also been proposed. A recent study showed significantly reduced inflammation and T-cell number in the dermis of *Nlrp10^−/−^* mice compared to wild-type (WT) controls during irritant-induced contact hypersensitivity (14). Others have suggested that *Nlrp10^−/−^* mice display an impaired T-cell immune response due to the inability of *Nlrp10^−/−^* DCs to transport antigens to draining lymph nodes (10, 11). However, the *Nlrp10^−/−^* mice used in these early studies carried an unintended mutation in *Dock8*, which was later found to be the primary cause of defective DC migration (15, 16). These reports do, however, highlight the need to further investigate the role of NLRP10 in adaptive immunity.

Here, we generated a novel *Nlrp10^−/−^* mouse line to investigate whether NLRP10 contributes to eliciting CD4^+^ T-cell-mediated immune response. We found that *Nlrp10^−/−^* DCs were defective in IL-12 production both *in vitro* and *in vivo* and had defective NF-κB signaling. Sub-optimal IL-12 synthesis impaired the production of IFNγ by antigen-specific CD4^+^ T cells, which diminished the ability of *Nlrp10^−/−^* mice to respond to *Mycobacterium tuberculosis* (*Mtb*) infection. These data suggest that NLRP10 is central to DC-mediated type 1 helper T (Th1)-cell-dependent immunity to intracellular bacteria.

## Materials and Methods

### Mice

The *Nlrp10^−/−^* mouse was generated by genome editing using CompoZr™ Zinc-Finger Nuclease technology (Sigma-Aldrich) (17–19). C57BL/6J mice were purchased from the Biological Resource Centre (BRC; A*STAR, Singapore). B6.129S7-*Rag1^tm1Mom^*Tg(TcraTcrb)425Cbn mice (OT-II-Rag1*^−/−^*, abbreviated to OT-II thereafter) were obtained from Taconic. All mice were maintained on a B6 background under specific pathogen-free conditions. For *Mtb* infection experiments, mice were housed in a Biosafety level 3 laboratory and treated humanely. All experimental procedures were approved by the Institutional Biosafety Committee (IBC) and Institutional Animal Care and Use Committee (IACUC) of the BRC (A*STAR) in compliance with their Guidelines for Animal Experiments.

### Cell Differentiation and Stimulation

Bone marrow (BM) cells were harvested from femurs and tibias of WT and *Nlrp10^−/−^* mice and cultured in Iscove’s Modified Dulbecco’s Medium (IMDM) with 10% fetal bovine serum (Hyclone), 1% penicillin/streptomycin, and 10% conditioned medium containing granulocyte-macrophage colony-stimulating factor for 7–8 days to generate DCs. Purity of the culture was assessed by flow cytometry, measuring the co-expression of CD11c and MHC-II. A total of 1 × 10^5^ DCs/well were plated in a 96-well plate and stimulated for 1, 2, and 3 days with the following TLR agonists: Pam(3)CSK(4) (1 µg/ml), LPS-B4 from *E. coli* (0.1 µg/ml), CpG DNA type B (0.001–10 µM), or PolyI:C (100 µg/ml), from InvivoGen. For CD40 stimulation, DCs were stimulated with MegaCD40L (50 ng/ml; Adipogen) for 8, 24, 48, and 72 h or with L929 cells expressing murine CD40 ligand (CD40L) at a DC:L929 ratio of 1:4 for 24 h.

### T-Cell Proliferation Assays

Lymph nodes and spleens of OT-II mice were harvested and processed to a single-cell suspension. Red blood cells (RBCs) were lysed in AKT buffer (0.83% NH_4_Cl, 0.1% KHCO_3_, 0.37 g EDTA), and the remaining cells were washed twice in PBS. CD4^+^ T cells were purified by magnetic bead immunoselection (Miltenyi Biotec) and labeled with CellTrace Violet (Life Technologies) in PBS for 15 min at 37°C before three washes with PBS. For *in vitro* experiments, DCs were incubated overnight with EndoGrade endotoxin-free ovalbumin (OVA) (20 µg/ml; Hyglos), then washed and plated in a 96-well plate at 5 × 10^4^/well with labeled OT-II CD4^+^ T cells (1 × 10^5^/well). After 3 days, cells were collected, washed, and labeled with anti-CD3, anti-CD4, anti-CD45.1, and anti-TCR Vβ5.1/5.2 antibodies (BD Bioscience). For *in vivo* experiments, CellTrace Violet-labeled OT-II CD4^+^ T cells were injected *via* the retro-orbital plexus vein into WT and *Nlrp10^−/−^* mice. After 24 h, mice were immunized by i.p. injection with EndoGrade endotoxin-free OVA protein (25 μg/mouse; Hyglos) mixed with 1 mg Imject Alum Adjuvant (Thermo Scientific) in 500 µl PBS. T-cell proliferation was measured by dye dilution 3 days later. Data were analyzed using FlowJo (TreeStar Inc.). The Division Index represents the average number of cell divisions of a cell in the original population, including the peak of undivided cells.

### DC and T-Cell Co-culture

Bone marrow-derived DCs (BMDCs) or conventional splenic CD11c^+^ MHC-II^high^ DCs were incubated overnight in EndoGrade endotoxin-free OVA (Hyglos) at the indicated concentrations. DCs were then washed, re-suspended in complete IMDM and replated at different quantities (1.25, 2.5, 5.0, or 10 × 10^4^) in 96-well plates in quadruplicates. Naive OVA-specific CD4^+^ T cells (1 × 10^5^/well) isolated from OT-II mouse spleens were added to each well. After 3 days, culture supernatants were harvested and analyzed for IFNγ by ELISA.

### Antigen Processing Assay

Bone marrow-derived DCs were pulsed for 1 h with 50 µg recombinant Eα-RFP (20). Cells were then washed in PBS and chased for the indicated time points. DCs were stained with the Y-Ae antibody [clone eBioY-Ae (YAe, Y-Ae); eBioscience] recognizing the Eα peptide 52-68 loaded on I-Ab MHC-II molecules and analyzed by flow cytometry.

### Bacterial Strains and Growth Conditions

*Mycobacterium tuberculosis* H37Rv was grown in Middlebrook 7H9 broth (BBL Microbiology Systems, USA) supplemented with Albumin Dextrose Catalase (Difco laboratories, USA) and 0.05% Tween 80 at 37°C for 5–7 days to an optical density of 0.4–0.5 (OD_600_). Mycobacterial cells were pelleted, re-suspended in fresh 7H9 broth with 20% glycerol and stored at −80°C. On the day of infection, the cells were thawed, washed, and sonicated before use.

### *Mtb* Murine Infection and Enumeration of *Mtb* Colony-Forming Unit (CFU)

Wild-type and *Nlrp10^−/−^* mice were infected with *Mtb* H37Rv using a nose-only aerosolization exposure system (CH Technologies, USA) as previously described (21). Mice (*n* = 3–4) were sacrificed on day 1 to determine the number of bacteria implanted in the lungs, or euthanized at pre-determined time points post-infection and the lungs were aseptically excised, washed in PBS and homogenized in PBS containing 0.25% Tween 80 using a MACS tissue dissociator (Miltenyi Biotech). The mycobacterial load in the lung tissue was quantified by plating dilutions of tissue homogenates on Middlebrook 7H11 agar plates supplemented with Oleic Albumin Dextrose Catalase (Sigma-Aldrich), in triplicate. Agar plates were incubated at 37°C for 2 weeks and visible colonies were counted. The CFU obtained from two or three dilutions was used to calculate the total number of CFUs per lung per mouse. The infection experiments were performed five times.

### Isolation and *Ex Vivo* Stimulation of Lung Cells from *Mtb*-Infected Mice

On day 35 post-infection, mice were euthanized and the lungs were aseptically removed and gently homogenized in PBS using a MACS tissue dissociator. Lung cells were passed through a 40 µm cell strainer (BD Biosciences). RBCs were lysed in RBC lysis buffer (Lonza), washed and re-suspended in RPMI-1640 medium and the cells were counted. Mouse BMDCs (5 × 10^6^) from WT and *Nlrp10^−/−^* mice were infected with *Mtb* (MOI 1). After 4 h, freshly isolated mouse lung cells (0.5 × 10^6^) from *Mtb*-infected *Nlrp10^−/−^* and WT mice were added and cultured for 16 h. Brefeldin A (1 µg/ml) and Monensin (1 µM) were added for the final 4 h of culture. The cells were then washed and stained with PE-Cy7-anti-CD3 (Biolegend #100220), Pacific-blue-anti-CD4 (Biolegend #100428), and LIVE/DEAD fixable green dead cell stain (Invitrogen #L23101). Cells were washed, fixed, and permeabilized using Cytofix/Cytoperm buffer (Cytofix/Cytoperm kit; BD Pharmingen), washed twice with Perm/wash buffer, and then stained with intracellular APC-anti-IFNγ (Biolegend #505810). Stained cells were fixed and analyzed using a BD LSR Fortessa™ (BD Biosciences). Data analysis was carried out using FlowJo.

### CpG DNA Treatment *In Vivo*

Mice were exposed to CpG DNA (ODN1826, class B, 50 μg/mouse, InvivoGen) by i.p. injection and plasma samples and spleens were collected 3 h later. Plasma was separated from the blood and cytokine levels were measured by ELISA. Total RNA was isolated from sorted splenic DCs and converted to cDNA by reverse transcription (Experimental Procedures in Supplementary Material).

To assess IFNγ production *in vivo*, OT-II CD4^+^ T cells were adoptively transferred to WT and *Nlrp10^−/−^* mice followed by i.p. injection of OVA (20 μg/mouse) and CpG DNA (20 μg/mouse) the following day. Spleens were harvested after 3 days and total splenocytes were co-cultured with WT and *Nlrp10^−/−^* BMDCs loaded or not with OVA. Cell-culture supernatants were collected after 3 days and assessed for IFNγ production by ELISA.

### Measurement of Cytokines

Mouse IL-12p40, IL-12p70, IL-6, IFNγ, and TNFα levels were measured using the Duoset ELISA kits (R&D Systems) according to the manufacturer’s instructions.

### Statistical Analysis

Data represent the mean ± SD or SE, as indicated. Data are derived from a minimum of three independent experiments unless stated otherwise. Statistical significance was determined by one-way ANOVA followed by Bonferroni *post hoc* test using GraphPad Prism 7 (GraphPad). A *p* < 0.05 was considered statistically significant.

## Results

### Characterization of *Nlrp10^−/−^* Mice

*Nlrp10^−/−^* mice were generated by CompoZr™ Zinc-Finger Nuclease technology (18) (Figures S1A–D in Supplementary Material) and mRNA expression analysis confirmed complete ablation of *Nlrp10* in the spleen and heart (Figure S1E in Supplementary Material). As a previous *Nlrp10^−/−^* mouse model (10) carried an unintended mutation in *Dock8* (15, 16), we specifically checked Dock8 expression in our *Nlrp10^−/−^* mice and found normal expression levels (Figure S1F in Supplementary Material). Our *Nlrp10^−/−^* mice were viable and fertile, developed a normal immune system composition (Figure S1G in Supplementary Material) and had a normal immune-cell profile in the spleen and lymph nodes when housed in a pathogen-free environment (Figure S1H in Supplementary Material).

### The NLRP3 Inflammasome Can Be Activated Independently of NLRP10

NLRP10 was suggested to negatively regulate the NLRP3 inflammasome activation in a glial cells line (22), but later studies using macrophages from *Nlrp10^−/−^* mice (carrying a *Dock8* deletion) (10, 15, 16) did not support these findings. Here, we used our *Nlrp10^−/−^* mice to examine the role of NLRP10 in inflammasome-mediated IL-1β release. LPS-primed, BM-derived *Nlrp10^−/−^* DCs produced normal levels of IL-1β in response to NLRP3 inflammasome activators, indicating that NLRP10 deficiency does not impact on NLRP3 inflammasome activation *in vitro* (Figure S2A in Supplementary Material). Moreover, monosodium urate-induced inflammation in *Nlrp10^−/−^* mice seemed normal, as evidenced by neutrophil and inflammatory monocyte recruitment to the peritoneal cavity (Figure S2B in Supplementary Material). These results indicate that NLRP10 is not involved in NLRP3 inflammasome activation or IL-1β production *in vitro* or *in vivo*.

### Absence of NLRP10 in DCs Impairs Antigen-Specific Secretion of IFNγ by CD4^+^ T Cells

**Figure 1:**
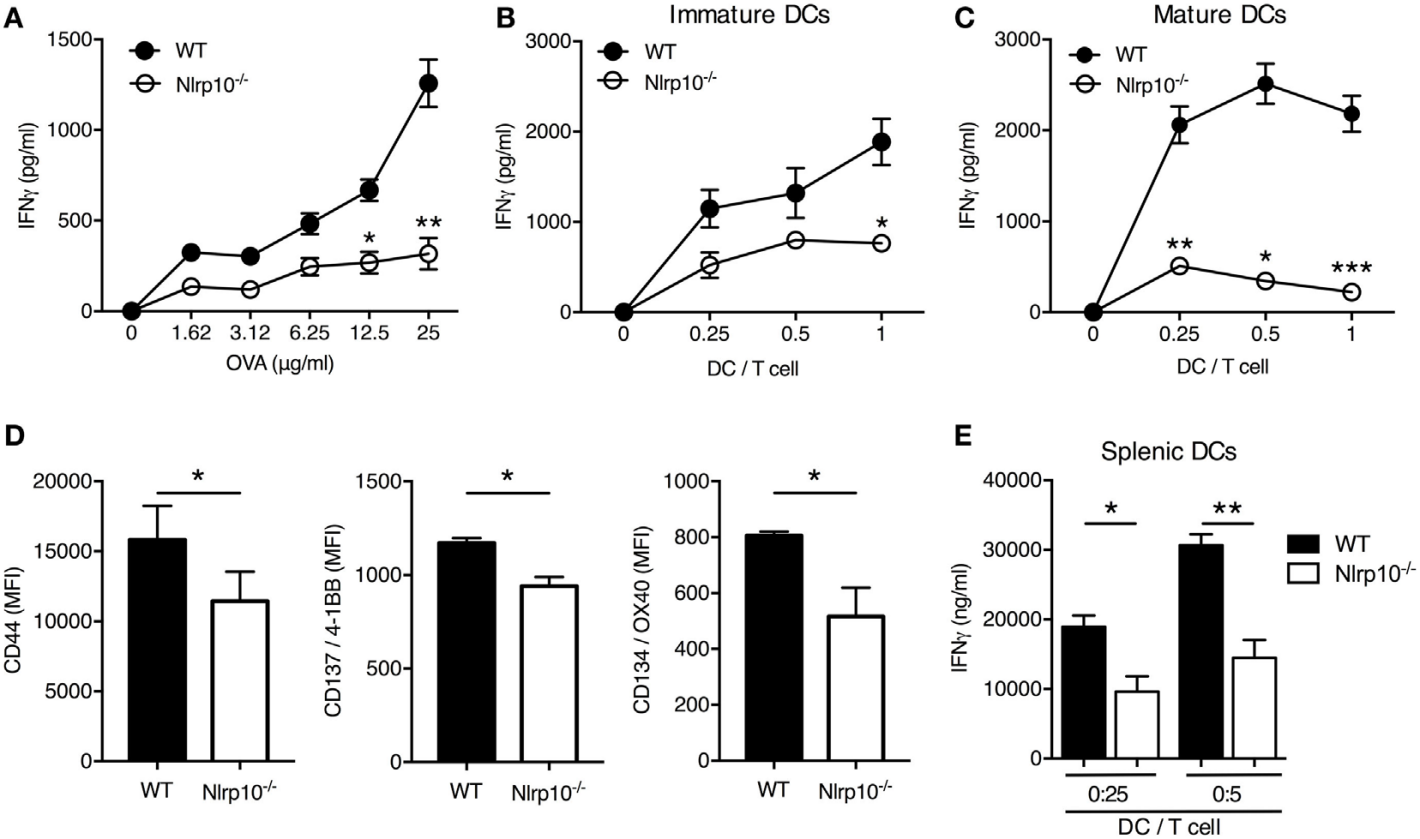
DC-mediated *Nlrp10* expression triggers IFNγ production by antigen-specific T cells. **(A–C)** IFNγ released by OT-II T cells co-cultured with immature **(A,B)** or mature **(C)** WT or *Nlrp10^−/−^* DCs loaded with increasing OVA concentrations (1.62–25 µg/ml) **(A)** and at different DC:OT-II T-cell ratios **(B,C)** measured in culture supernatants after 3 days by ELISA. Data presented are from single experiments and are representative of three independent experiments. **(D)** Expression of T-cell activation markers 3 days after incubation with OVA-loaded WT or *Nlrp10^−/−^* DCs. **(E)** IFNγ production by OT-II T cells co-cultured for 3 days with different amounts of splenic DCs isolated from WT or *Nlrp10^−/−^* mice and loaded overnight with OVA antigen (25 µg/ml). Data are expressed as the means ± SD. *p < 0.05; **p < 0.01; ***p < 0.001. Abbreviations: DC, dendritic cells; WT, wild-type; MFI, median fluorescence intensity; OVA, ovalbumin.

As DCs are central initiators of the T-cell response, we assessed *Nlrp10* expression in conventional splenic CD8a^+^ DCs, which could be induced upon CpG DNA stimulation (Figures S3A,B in Supplementary Material), suggesting a role for *Nlrp10* in DC function. To further interrogate this finding, we used OVA-specific OT-II T cells derived from OT-II/*Rag1^−/−^* mice. *Nlrp10^−/−^* DCs elicited a low CD4^+^ T-cell activation, as inferred by IFNγ production, in a dose response manner to OVA (Figure 1A) and at all DC:OT-II T-cell ratios tested (Figure 1B). The inability of *Nlrp10^−/−^* DCs to trigger an OVA-specific CD4^+^ T-cell response was independent of the maturation stage of the DCs, as antigen-activated OT-II CD4^+^ T cells stimulated with LPS-matured *Nlrp10^−/−^* DCs released lower levels of IFNγ compared to T cells cultured with matured WT DCs (Figure 1C). Additionally, OT-II CD4^+^ T cells co-cultured with OVA-pulsed *Nlrp10^−/−^* DCs expressed lower amounts of T-cell activation markers, including CD44, CD137/4-1BB, and CD134/OX40, compared to OT-II CD4^+^ T cells co-cultured with WT DCs (Figure 1D). A similar defect in DC-mediated IFNγ production from OT-II CD4^+^ T cells was observed in *Nlrp10^−/−^* splenic DCs (Figure 1E), indicating that this effect is not restricted to *in vitro* BMDCs but extends to *ex vivo* isolated primary DCs. Collectively, these findings indicate that DC NLRP10 expression contributes to CD4^+^ T-cell activation.

### *Nlrp10^−/−^* DCs Exhibit Normal Antigen Uptake, Processing, and Presentation and Elicit CD4^+^ T-Cell Proliferation

**Figure 2:**
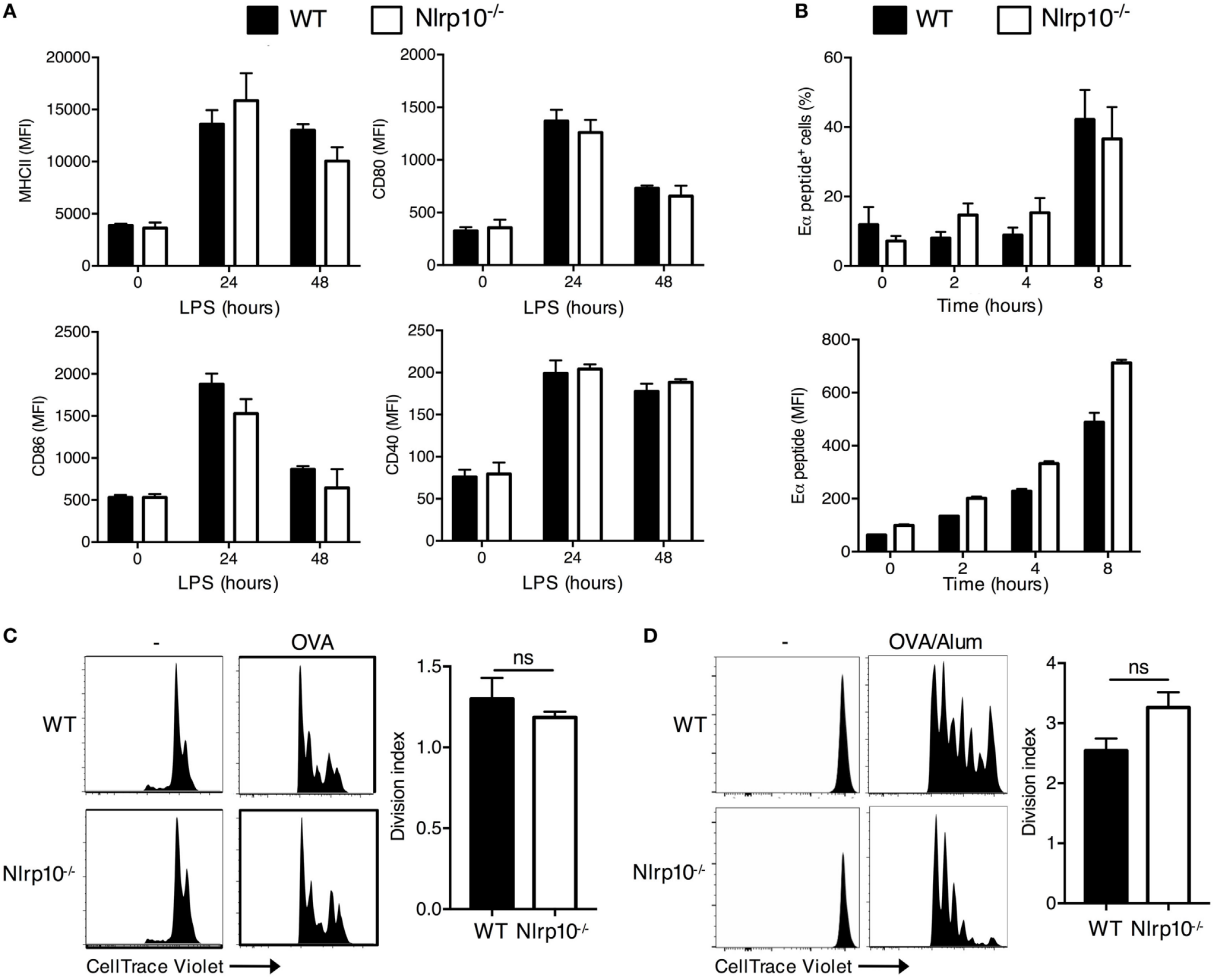
*Nlrp10^−/−^* dendritic cells (DCs) show normal antigen uptake, processing and presentation, and T-cell stimulatory activity. **(A)** Cell-surface expression of MHC-II and co-stimulatory markers CD80, CD86, and CD40 in WT and *Nlrp10^−/−^* DCs were analyzed at steady state and after LPS treatment by flow cytometry. Each column represents the mean ± SD of three independent experiments. **(B)** WT and *Nlrp10^−/−^* DCs were loaded with Eα protein (50 µg) for 1 h; cell-surface presentation of Eα peptide (52–68) expressed as a percentage and MFI was assessed at the indicated time points by flow cytometry. **(C)** Proliferation of CellTrace Violet-labeled OT-II CD4^+^ T cells co-cultured with WT or *Nlrp10^−/−^* DCs loaded with OVA (20 µg/ml) assessed by CellTrace Violet dilution by flow cytometry. **(D)** Proliferation of CellTrace Violet-labeled OT-II CD4^+^ T cells that were adoptively transferred into WT or *Nlrp10^−/−^* mice immunized with OVA (25 μg/mouse) in Alum adjuvant. Proliferation was measured 3 days after immunization. Graphical representation of the CellTrace Violet dilution and consolidated division indices for inguinal lymph nodes are shown. Data are expressed as the means ± SD. *n* = 4 WT and *n* = 5 *Nlrp10^−/−^* mice per group. *p < 0.05; ns, not significant. Abbreviations: Alum, aluminum hydroxide; LN, lymph node; LPS, lipopolysaccharide; MFI, median fluorescence intensity; OVA, ovalbumin; WT, wild-type.

We next sought to determine the mechanisms underlying the sub-optimal antigen-specific T-cell response elicited by *Nlrp10^−/−^* DCs. We first assessed whether *Nlrp10* deficiency resulted in aberrant expression of antigen-presenting MHC-II or co-stimulatory molecules on DCs. We noticed that the steady state, LPS-induced or CpG-induced expression of MHC-II, CD80, CD86, and CD40 was equivalent between *Nlrp10^−/−^* and WT DCs (Figure 2A; Figure S4 in Supplementary Material). Next, we determined whether NLRP10 influences the presentation of cognate peptides to naive antigen-specific CD4^+^ T cells. WT and *Nlrp10^−/−^* DCs were pulsed with the Eα protein for 1 h, and the presence of processed Eα peptide 52-68 bound to I-Ab MHC-II molecules was assessed at different time points. Total Eα peptide and the percentage of DCs positive for the Eα were similar between WT and *Nlrp10^−/−^* DCs (Figure 2B), suggesting that NLRP10 is dispensable for antigen processing and presentation. We then examined the uptake of florescent OVA by *Nlrp10^−/−^* DCs and their migration to the draining lymph node *in vivo*. The number of CD103^+^ and CD11b^+^ DCs, as well as uptake of OVA-AF647 by these DC subsets in *Nlrp10^−/−^* mice, was similar to that of WT mice, again indicating no defect in antigen uptake or DC migration *in vivo* (Figure S5 in Supplementary Material). These findings are consistent with another study using a *Nlrp10^−/−^* mouse model with normal *Dock8* expression (16).

Finally, we examined whether loss of NLRP10 impairs the ability of DCs to elicit CD4^+^ T-cell proliferation. WT and *Nlrp10^−/−^* DCs were primed with OVA and co-cultured with CellTrace Violet-labeled CD4^+^ OT-II T cells, and the dilution of the violet dye was measured after 3 days by flow cytometry. Here, *Nlrp10^−/−^* DCs elicited normal OVA-specific T-cell proliferation *in vitro* (Figure 2C). To assess CD4^+^ T-cell proliferation *in vivo*, WT and *Nlrp10^−/−^* mice underwent adoptive transfer with CellTrace Violet-labeled CD4^+^ OT-II T cells before immunization with OVA plus alum. The division of OT-II T cells in the inguinal lymph node was determined 5 days after immunization. *Nlrp10* deficiency had no marked effect on the OVA-specific proliferation of OT-II CD4^+^ T cells in the draining lymph nodes of *Nlrp10^−/−^* mice (Figure 2D). These results indicate that although NLRP10 in DCs is dispensable for responding CD4^+^ T cells to promote proliferation, it is absolutely required to elicit optimal IFNγ production.

### *Nlrp10^−/−^* DCs Produce Less IL-12 in Response to CD40 Ligation

**Figure 3:**
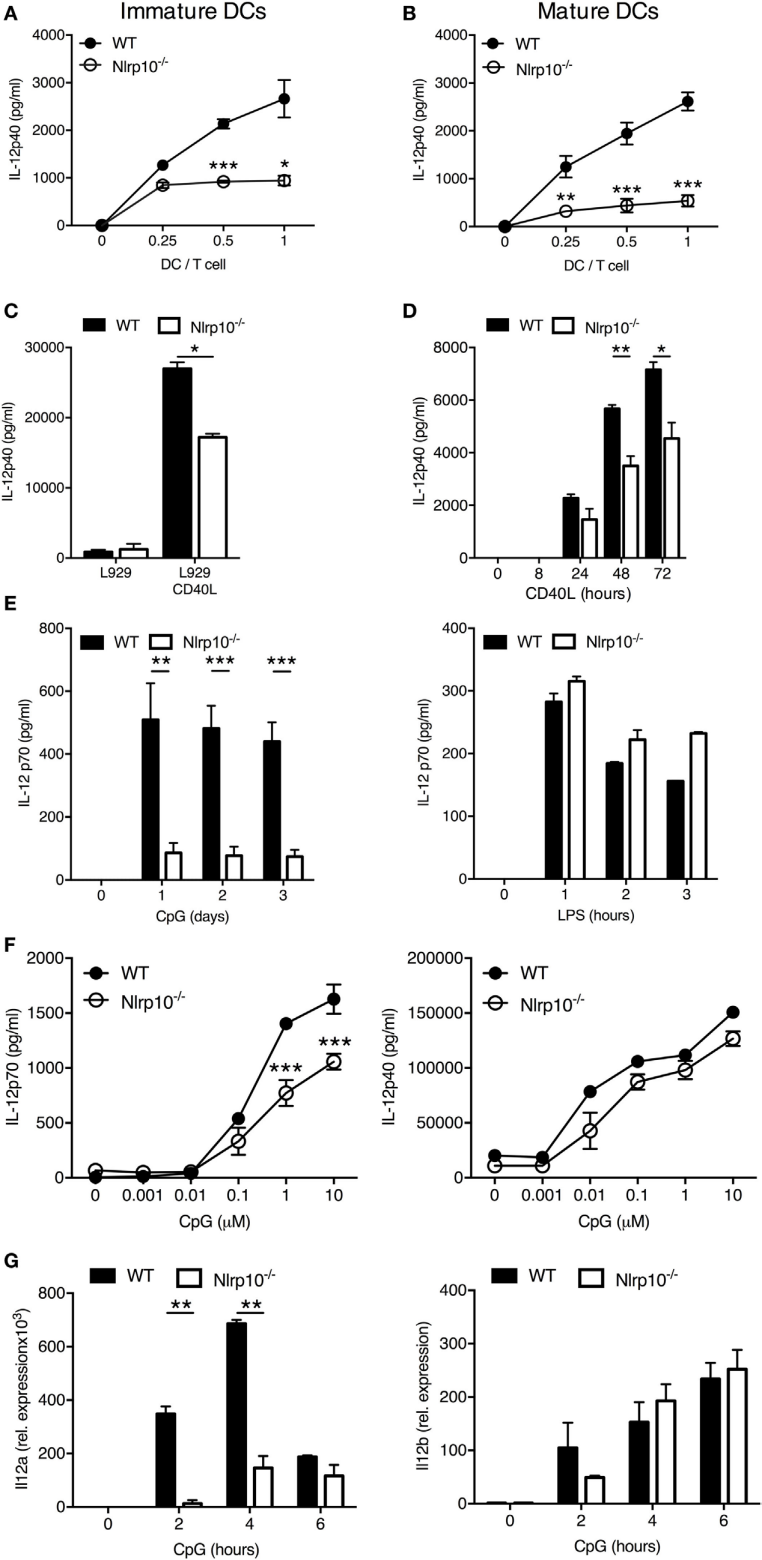
*Nlrp10^−/−^* DCs exhibit impaired IL-12 production after CD40 ligation or CpG DNA stimulation. **(A,B)** ELISA measurement of IL-12p40 levels in cell supernatants from OT-II CD4^+^ T cells co-cultured with immature **(A)** or matured **(B)** WT or *Nlrp10^−/−^* DCs loaded with ovalbumin (20 µg/ml) for 3 days. **(C)** WT and *Nlrp10^−/−^* DCs were incubated with L929 fibroblasts expressing or not expressing CD40 ligand (CD40L). Cell supernatants were collected after 3 days and the IL-12p40 levels were measured by ELISA. **(D)** ELISA measurement of IL-12p40 production by WT and *Nlrp10^−/−^* DCs stimulated by MegaCD40L for the indicated times. **(E)** WT and *Nlrp10^−/−^* DCs were left untreated or stimulated with either CpG DNA or LPS and the IL-12p70 levels in supernatants were then determined at the indicated times. **(F)** ELISA measurements of IL-12p70 and IL-12p40 production by WT and *Nlrp10^−/−^* DCs stimulated for 12 h with increasing concentrations of CpG DNA (0.001–10 µM). **(G)** Relative expression of *Il12a* and *Il12b* in WT and *Nlrp10^−/−^* DCs stimulated with CpG DNA (1 µM) for the indicated times and assessed by semi-quantitative RT-PCR. Data shown represent the mean ± SD and represent an experiment performed in triplicates and repeated with at least with three different DC preparations. *p < 0.05; **p < 0.01; ***p < 0.001. Abbreviations: DCs, dendritic cells; LPS, lipopolysaccharide; WT, wild-type.

We next aimed to delineate the mechanism(s) underlying diminished IFNγ production by CD4^+^ T cells upon *Nlrp10^−/−^* DC stimulation. To this aim, we assessed the production of IL-12 by DCs, which is necessary for optimal IFNγ production and induction of Th1 cell responses (23–25). OVA-primed, immature *Nlrp10^−/−^*, and WT DCs were incubated at different DC:T-cell ratios with OT-II CD4^+^ T cells for 3 days before measuring the production of IL-12p40 in the culture supernatant. Robust IL-12p40 production was found in the OT-II T-cell and WT DC co-culture, whereas significantly less IL-12p40 was found in the OT-II T-cell and *Nlrp10^−/−^* DC co-culture (Figure 3A). A similar defect in IL-12p40 production was observed in matured *Nlrp10^−/−^* DCs compared to WT DCs (Figure 3B).

IL-12p40 production can be elicited when DCs interact with T cells *via* CD40 cross-linking with the CD40L (26). Therefore, we investigated the response of DCs to CD40 ligation by co-culturing them with L929 fibroblasts transfected with CD40L. WT DCs stimulated with CD40L-transfected fibroblasts produced high levels of IL-12p40, whereas their co-culture with mock-transfected fibroblasts had no effect (Figure 3C). Stimulation of *Nlrp10^−/−^* DCs with CD40L-transfected fibroblasts resulted in significantly less IL-12p40 production compared to WT DCs (Figure 3C). Similarly, upon ligation of CD40 with recombinant MegaCD40L (that simulates CD40L aggregates) *Nlrp10^−/−^* DCs produced significantly less IL-12p40 compared to WT DCs (Figure 3D). Of note, stimulation of DCs in culture with antigen-activated T cells or CD40L failed to induce detectable levels of IL-12p70 (data not shown), as previously reported (27). These results indicate that NLRP10 is important for optimal IL-12p40 production after CD40 ligation on DCs.

### NLRP10 Differentially Modulates IL-12 Production in Response to TLR Stimulation

A microbial stimulus is required for effective production of active IL-12p70 heterodimers by DCs (27). This effect is mostly due to the rapid upregulation of CD40 on DCs by microbial stimuli, including TLR agonists (28). Microbial stimulation alone can also elicit IL-12p70 production by DCs (29). We thus investigated the ability of *Nlrp10^−/−^* DCs to produce IL-12p40 and IL-12p70 in response to TLR stimulation. WT and *Nlrp10^−/−^* DCs were stimulated with Pam(3)CSK(4) (TLR1/2 agonist), LPS (TLR4 agonist), CpG DNA (TLR9 agonist), or PolyI:C (TLR3 agonist) before assessment of IL-12p40 production. With the exception of PolyI:C, all tested TLR agonists elicited the production of normal IL-12p40 levels by *Nlrp10^−/−^* DCs (Figures S6A,B in Supplementary Material). However, at the doses and times tested in our study, *Nlrp10^−/−^* DCs released significantly less IL-12p70 in response to CpG DNA compared to WT DCs, but no significant difference was observed between WT and *Nlrp10^−/−^* DCs in response to LPS (Figure 3E). IL-12p70 secretion by DCs in response to PolyI:C stimulation was undetectable (data not shown). Lower levels of IL-12p70 released by *Nlrp10^−/−^* DCs were also evident at different CpG DNA concentrations compared to WT DCs (Figure 3F). However, no significant difference in the levels of IL-12p40 was observed between WT and *Nlrp10^−/−^* DCs following stimulation with CpG DNA (Figure 3F; Figure S4 in Supplementary Material). We also examined the expression of *Il12a* and *Il12b* genes that encode IL-12p35 and IL-12p40, respectively. While *Il12b* transcription was normal (similar to normal IL-12p40 protein), *Il12a* expression was significantly diminished in *Nlrp10^−/−^* DCs compared to WT DCs in response to CpG DNA stimulation (Figure 3G). Similar to IL-12, the production of IL-6 and TNFα by CpG DNA or PolyI:C-stimulated *Nlrp10^−/−^* DCs was also diminished compared to WT DCs (Figure S6C in Supplementary Material). Taken together, these results indicate the critical involvement of NLRP10 in the differential control of TLR9-mediated cytokine production in response to CpG DNA stimulation by DCs.

### *Nlrp10^−/−^* DCs Display Attenuated NF-κB Signaling

**Figure 4:**
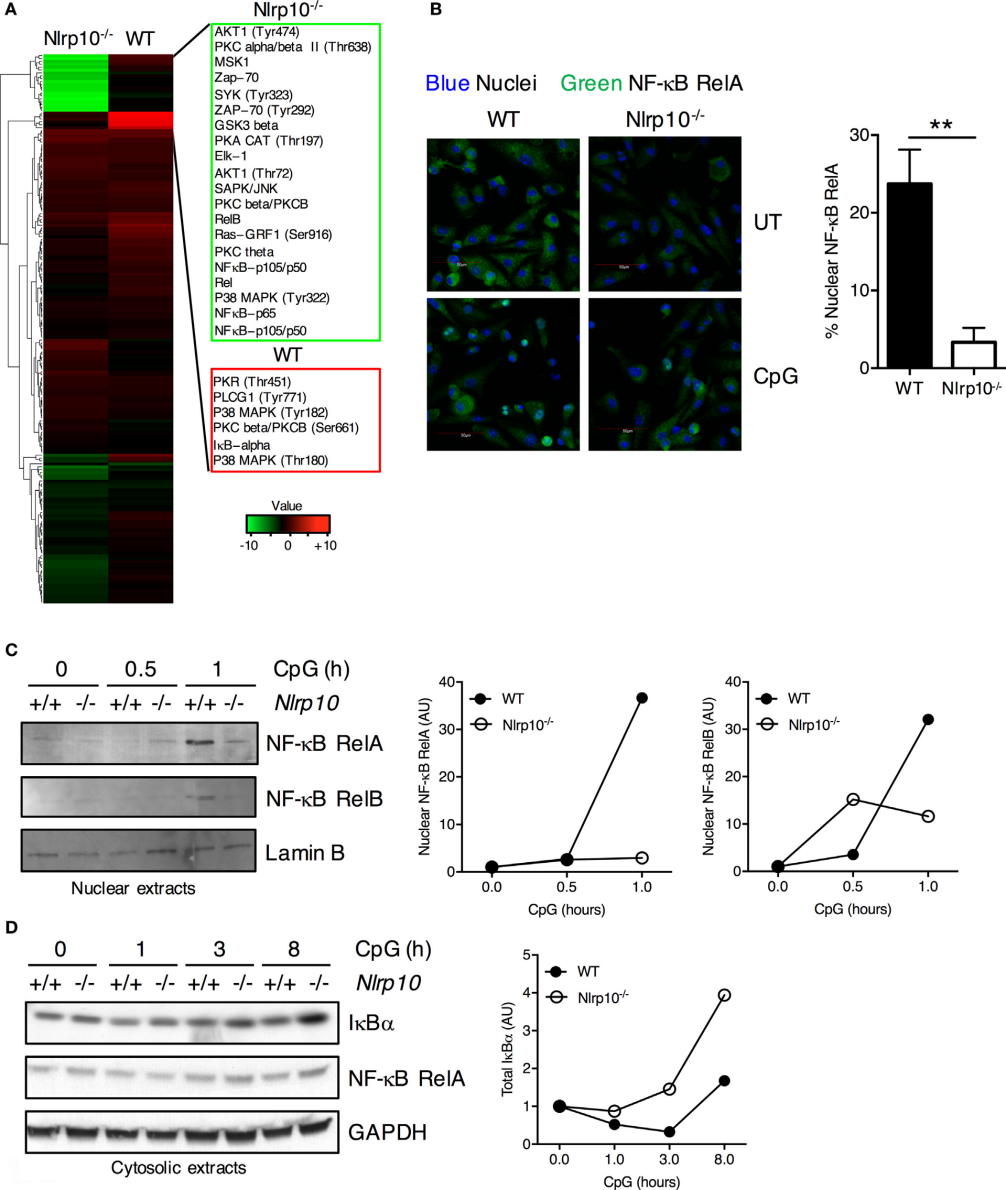
*Nlrp10^−/−^* dendritic cells (DCs) elicit sub-optimal NF-κB activation following CpG DNA stimulation. **(A)** Heat map of log2 fold changes for *Nlrp10^−/−^* and WT DCs after CpG DNA treatment for 45 min (upregulated expression after treatment in red and downregulated expression in green). The proteins are clustered using hierarchical clustering with Euclidean distance. **(B)** Nuclear translocation of NF-κB RelA subunit in WT and *Nlrp10^−/−^* DCs following 30 min stimulation with CpG DNA. Confocal microscopic images show NF-κB RelA (green) and nuclei (blue) (magnification 40×; scale bars represent 50 µm). Percentage of DCs with predominantly nuclear NF-κB RelA in CpG DNA-treated WT and *Nlrp10^−/−^* DCs is shown on the right. Data represent the mean ± SD. **(C,D)** WT and *Nlrp10^−/−^* DCs were treated with CpG DNA for the indicated times. NF-κB RelA was examined in both the nuclear and cytosolic lysates, whereas NF-κB RelB and total IκBα were examined in the nuclear and cytosolic lysates, respectively. Lamin B and GAPDH were used as loading controls and as nuclear and cytosolic markers, respectively. Densitometry analysis of NF-κB RelA, RelB, and total IκBα. Data shown are representative of at least three independent experiments. **p < 0.01. Abbreviations: AU, arbitrary units; UT, untreated; WT, wild-type.

Decreased cytokine production observed in *Nlrp10^−/−^* DCs may be due to reduced activation of inflammatory signaling pathways. To elucidate the molecular mechanism by which NLRP10 regulates signal transduction, we carried out a high-throughput proteomic screen of potential NLRP10 targets using antibody arrays. NF-κB RelA and RelB, IκBα, and phosphorylated p38 MAPK levels were markedly diminished in *Nlrp10^−/−^* DCs compared to WT DCs after CpG DNA stimulation (Figure 4A; Supplementary Table 1), indicating a role for NLRP10 in promoting NF-κB activity in DCs.

To validate the changes in NF-κB protein levels observed in our antibody array analysis, we measured the nuclear translocation of RelA (NF-κB p65) in WT and *Nlrp10^−/−^* DCs following CpG DNA stimulation. Consistently, RelA nuclear translocation was significantly decreased in *Nlrp10^−/−^* DCs compared to WT (Figures 4B,C). Reciprocally, the degradation of the NF-κB inhibitor IκBα following CpG DNA stimulation was increased in *Nlrp10^−/−^* DCs (Figure 4D). CpG DNA stimulation of TLR9 can induce non-canonical activation of NF-κB. Consequently, we explored the role of NLRP10 in the nuclear translocation of RelB—the “non-canonical” NF-κB subunit—and found that its nuclear translocation was also diminished in *Nlrp10^−/−^* DCs (Figure 4C). Taken together, these data indicate a reduced activation of NF-κB pathway in *Nlrp10^−/−^* DCs following CpG DNA stimulation.

### CpG DNA-Induced Th1 Response Is Abolished in *Nlrp10^−/−^* Mice

**Figure 5:**
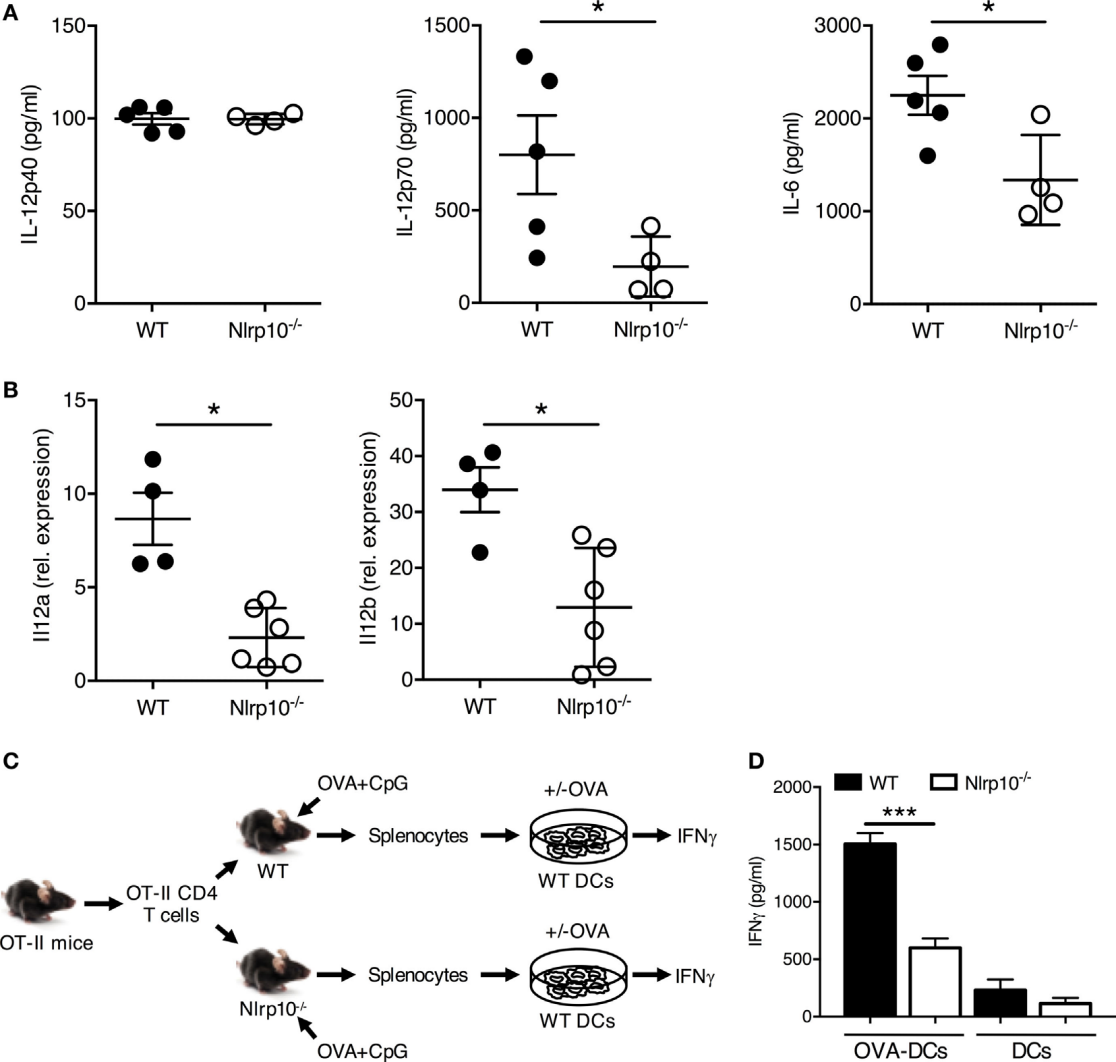
*Nlrp10^−/−^* T cells show impaired IL-12 production and IFNγ release *in vivo* in response to TLR9 stimulation. **(A)** IL-12p40, IL-12p70 and IL-6 levels were measured in the sera of WT and *Nlrp10^−/−^* mice treated with CpG DNA for 3 h. Data are expressed as the mean ± SD. **(B)** Expression of *Il12a* and *Il12b* in splenic DCs isolated from WT and *Nlrp10^−/−^* mice 3 h after CpG DNA treatment. **(C,D)** Schematic of the adoptive transfer protocol. WT and *Nlrp10^−/−^* mice were adoptively transferred with CD4^+^ OT-II T cells and then exposed 24 h later to OVA plus CpG DNA *via* intraperitoneal injection. IFNγ production by OT-II T cells was evaluated by ELISA after *in vitro* re-stimulation for 3 days with WT DCs loaded or not with OVA. *p < 0.05; ***p < 0.001. Abbreviations: DCs, dendritic cells; OVA, ovalbumin; WT, wild-type.

To confirm the impaired ability of *Nlrp10^−/−^* DCs to elicit cytokine production *in vivo*, we exposed WT and *Nlrp10^−/−^* mice to CpG DNA or LPS *via* intraperitoneal (i.p.) injection, and measured the plasma levels of IL-12p40, IL-12p70, and IL-6 after 3 h. While CpG DNA-induced plasma IL-12p40 levels were comparable between *Nlrp10^−/−^* and WT mice, IL-12p70 and IL-6 productions were significantly lower in *Nlrp10^−/−^* mice compared to WT (Figure 5A). By contrast, LPS treatment induced comparable IL-12p40, IL-12p70, and IL-6 production between WT and *Nlrp10^−/−^* mice (Figure S7 in Supplementary Material). In addition, CpG DNA-induced transcription of *Il12a* and *Il12b in vivo* was significantly reduced in splenic DCs isolated from *Nlrp10^−/−^* mice compared to DCs from WT (Figure 5B).

Next, we aimed to determine whether reduced IL-12 production by DCs from *Nlrp10^−/−^* mice upon CpG DNA exposure could promote defective IFNγ production by CD4^+^ T cells in an antigen-specific setting. OT-II CD4^+^ T cells were adoptively transferred intravenously into WT and *Nlrp10^−/−^* mice prior to i.p. injection of OVA and CpG DNA. On day 3, CD4^+^ T cells were isolated and cultured with WT DCs that were loaded or not with OVA, and IFNγ production was analyzed (Figure 5C). OT-II CD4^+^ T cells from *Nlrp10^−/−^* mice produced less IFNγ compared to the similar cells from WT mice upon *in vitro* re-stimulation with OVA-loaded WT DCs (Figure 5D). Reduced IFNγ production from co-cultures of OVA-loaded WT DCs with OT-II CD4^+^ T cells isolated from *Nlrp10^−/−^* mice was not due to a diminished proportion of OT-II CD4^+^ T cells within the total CD4^+^ T-cell population, as the percentage of OT-II CD4^+^ T cells in the spleens of WT and *Nlrp10^−/−^* mice were similar (data not shown). These data demonstrate that NLRP10 modulates the production of high levels of bioactive IL-12p70, upon exposure to CpG DNA *in vivo*, which leads to optimal IFNγ production by CD4^+^ T cells.

### Impaired IL-12 Production Renders *Nlrp10^−/−^* Mice Susceptible to *Mtb* Infection

**Figure 6:**
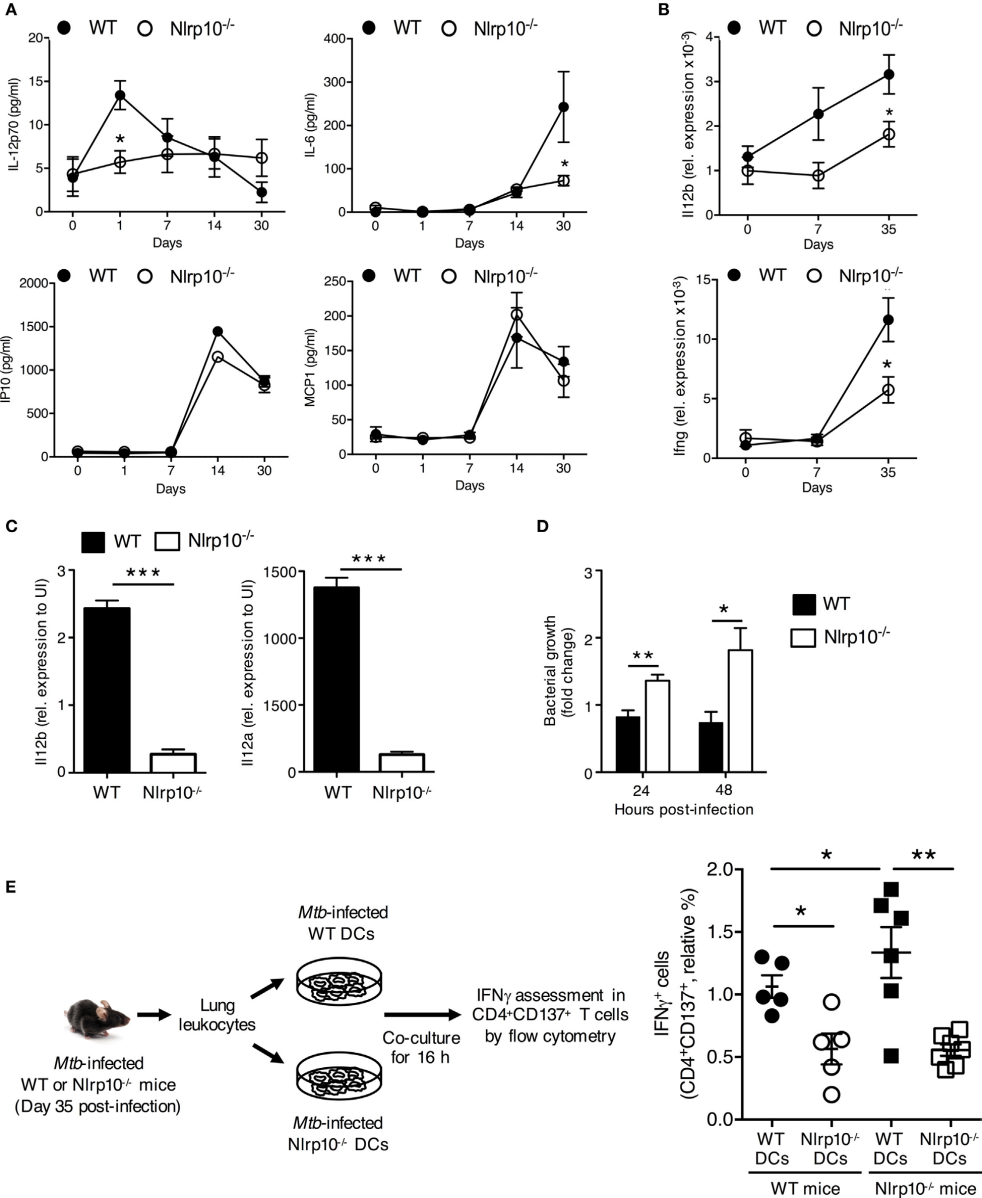
*Nlrp10^−/−^* DCs have attenuated capacity to stimulate a *Mtb*-specific CD4^+^ T-cell response. **(A)** IL-12p70, IL-6, IP10 and MCP1 levels in lung homogenates of WT and *Nlrp10^−/−^* mice at different times after *Mtb* infection, as determined by ELISA. **(B)** *Il12b* and *Ifng* mRNA expressions in lung leukocytes isolated from *Mtb*-infected WT and *Nlrp10^−/−^* mice, as determined by semi-quantitative RT-PCR. **(C)** *Il12a* and *Il12b* mRNA expressions in bone marrow-derived dendritic cells (BMDCs) infected with *Mtb*, as assessed by semi-quantitative RT-PCR. **(D)** CFUs measured in *Nlrp10^−/−^* and WT BM-derived DCs 24 and 48 h after *Mtb* infection. **(E)** Lung leukocytes collected from *Nlrp10^−/−^* and WT mice 35 days post-*Mtb* infection were re-stimulated *in vitro* with *Mtb*-infected WT or *Nlrp10^−/−^* DCs for 16 h. The percentage of IFNγ-producing CD4^+^CD137^+^ T cells was assessed by flow cytometry. Data represent the mean ± SE. *p < 0.05; **p < 0.01; ***p < 0.001. Abbreviations: DCs, dendritic cells; FACS, florescence-activated cell sorting; *Mtb, Mycobacterium tuberculosis*; WT, wild-type; CFUs, colony-forming units.

IL-12 and IFNγ are essential for protective cell-mediated immunity against various intracellular bacteria in mice (30) and in humans (31, 32). We thus sought to determine whether NLRP10 is essential for immunity to *Mtb*–the etiological agent of tuberculosis. WT and *Nlrp10^−/−^* mice were infected with *Mtb via* aerosol inhalation, and the expression of cytokines, the phenotype of myeloid cells, and the bacterial load in the lung were investigated. *Mtb*-infected *Nlrp10^−/−^* mice had significantly lower levels of active IL-12p70 and IL-6 cytokines (Figure 6A) and expressed reduced *Il12b* and *Ifnγ* (Figure 6B), in the lung compared to WT mice. However, IP10 and MCP1 chemokine levels were equivalent between WT and *Nlrp10^−/−^* mice (Figure 6A). In addition, *Mtb*-induced transcription of *Il12a* and *Il12b in vitro* was significantly reduced in BMDCs from *Nlrp10^−/−^* mice compared to BMDCs from WT mice (Figure 6C). Reduced IL-12 production by *Nlrp10^−/−^* BMDCs was not due to reduced *Mtb* infection, since bacterial counts were significantly increased in *Nlrp10^−/−^* BMDCs compared to WT BMDCs (Figure 6D). These data indicate that the increased survival of *Mtb* in *Nlrp10^−/−^* DCs is probably due to reduced killing effector functions in NLRP10-deficient DCs (Figure 6D).

To determine whether reduced DC-derived IL-12 results in decreased IFNγ production by CD4^+^ T cells, total lung leukocytes from *Mtb*-infected *Nlrp10^−/−^* and WT mice were re-stimulated *ex vivo* with *Mtb*-infected WT or *Nlrp10^−/−^* DCs (Figure 6E). CD4^+^ T cells from *Mtb*-infected WT mice stimulated with *Nlrp10^−/−^* DCs produced lower levels of IFNγ compared to those stimulated with WT DCs (Figure 6E). In addition, CD4^+^ T cells from *Mtb*-infected *Nlrp10^−/−^* mice when re-stimulated with WT DCs restored the IFNγ production (Figure 6E). Collectively, these data indicate the attenuated capacity of *Nlrp10^−/−^* DCs to stimulate an *Mtb*-specific CD4^+^ T-cell response and that NLRP10 deficiency in CD4^+^ T cells does not impact on the ability of these cells to produce IFNγ.

**Figure 7:**
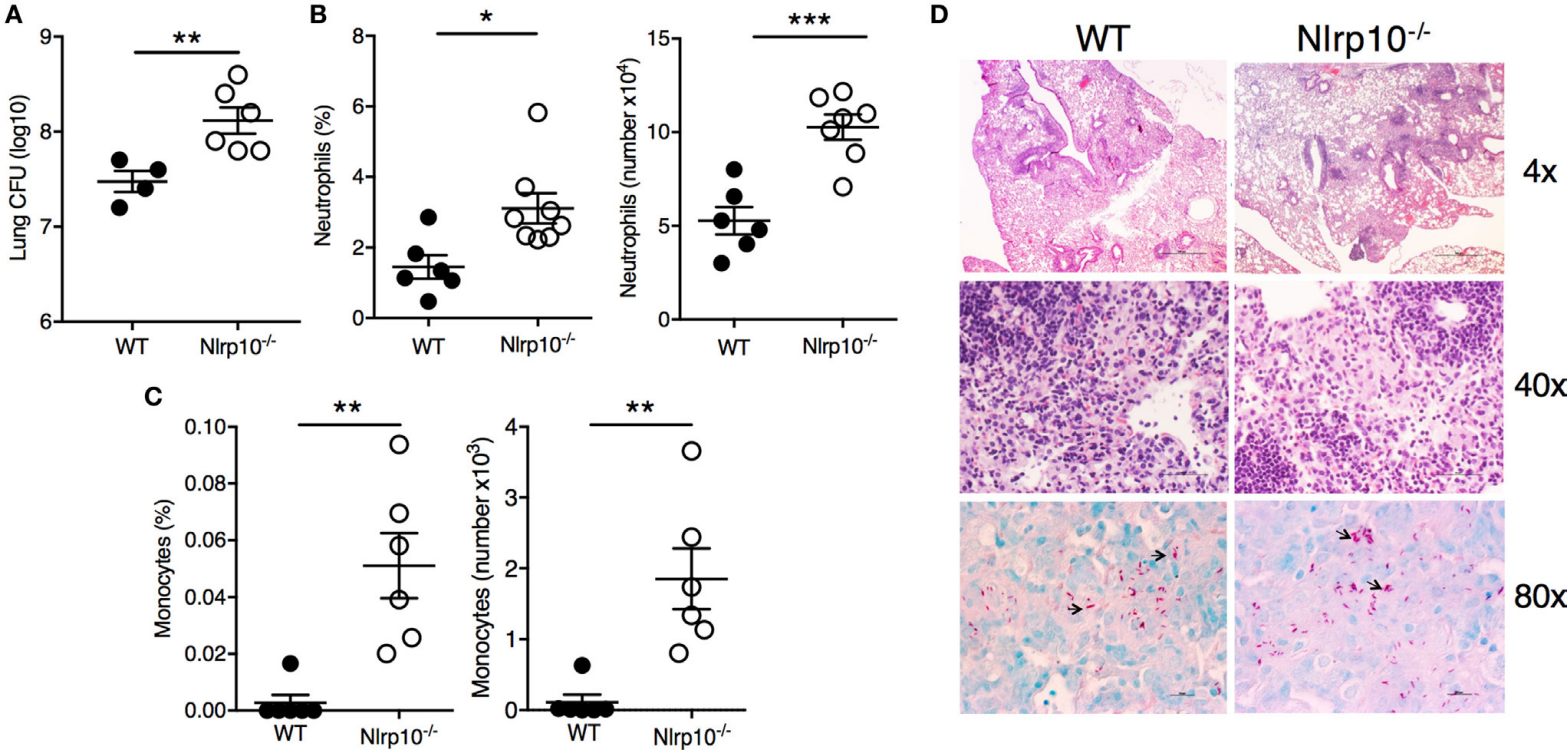
*Nlrp10* deficiency is associated with increased susceptibility to *Mtb* infection. **(A)** Bacterial load was measured in lung from *Nlrp10^−/−^* and WT mice 35 days after *Mtb* infection. Percentage of CD11b^+^Ly6G^+^ neutrophils **(B)** and CD11b^+^Ly6C^+^ inflammatory monocytes **(C)** was determined by flow cytometry in lung cellular suspensions obtained from *Nlrp10^−/−^* and WT mice 7 days after *Mtb* infection. Frequency and absolute cell numbers are represented as mean ± SE. **(D)** Representative lung sections of *Nlrp10^−/−^* and WT mice infected with *Mtb* for 30 days. Magnifications 4×, 40× (H&E), and 80× (AF). *p < 0.05; **p < 0.01; ***p < 0.001. Abbreviations: CFUs, colony-forming units; WT, wild-type.

The impairment in cytokine production (Figure 6) in *Nlrp10^−/−^* mice was associated with a higher (~1 log_10_) bacillary burden in the lungs of *Nlrp10^−/−^* mice compared to WT mice (Figure 7A). This was accompanied by pronounced accumulation of CD11b^+^Ly6G^+^ neutrophils (Figure 7B) and inflammatory monocytes (CD11b^+^Ly6C^+^; Figure 7C) in the lungs of *Mtb*-infected *Nlrp10^−/−^* mice. Interestingly, a histologic examination of the lungs from WT and *Nlrp10^−/−^* mice 30 days post-infection did not reveal any striking differences in terms of the cellular diversity and composition of the lesions (Figure 7D). Lungs from WT and *Nlrp10^−/−^* mice displayed diffuse coalescent lesions, with numerous infiltrating macrophages and lymphocytes. However, higher numbers of intracellular acid-fast bacilli were observed in *Nlrp10^−/−^* mice compared to WT. Taken together, these observations indicate that NLRP10 mediates the ability of lung mononuclear phagocytes to produce IL-12, which drives a protective Th1-cell-mediated immune response that is critical for protection during *Mtb* infection.

## Discussion

To date, the physiological role of NLRP10 in DCs and the immune response has been largely uncharacterized, mainly due to the lack of a suitable animal model. Here, we generated a new *Nlrp10^−/−^* mouse to demonstrate *in vitro* and *in vivo* that NLRP10 has an important role in inducing IL-12 production by DCs. Sub-optimal IL-12 production by *Nlrp10^−/−^* DCs resulted in impaired IFNγ production by antigen-specific CD4^+^ T cells, which possibly leads to increased susceptibility to *Mtb* infection. Our data emphasize the protective nature of NLRP10 against microbial pathogens, thus adding NLRP10 to the group of NLR family members such as NOD1, NOD2, NLRC4, NLRP3, and NLRP12 that initiate pro-inflammatory signaling to mediate host resistance toward bacterial infections (6, 33–35).

IL-12-induced IFNγ is primarily produced by T cells and has an essential role in the host defense against various bacterial pathogens, including *Mtb*. Mice deficient in *Il12, Il12R*, genes regulating IFNγ expression (i.e., *Tbet* and *Stat1*) or IFNγ itself are extremely susceptible to *Mtb* infection (30, 36, 37). Furthermore, people with inborn mutations in genes within the IL-12/IFNγ axis are highly susceptible to normally avirulent, non-tuberculous mycobacterial infections (38). Here, we found that *Mtb*-infected *Nlrp10^−/−^* mice had attenuated IL-12p70 production, which is due in part to the impaired ability of *Nlrp10^−/−^*DCs to synthesize *Il12a* and *Il12b*. Reduced IL-12p70 release resulted in diminished production of IFNγ by CD4^+^ T cells. The results of our criss-cross experiment demonstrated that NLRP10 has an important role in DCs, but not T cells, during *Mtb* infection, as the percentage of IFNγ-producing CD4^+^ T cells was restored when CD4^+^ T cells from *Nlrp10^−/−^* mice were re-stimulated *ex vivo* by *Mtb*-infected WT DCs, but not by DCs obtained from *Nlrp10^−/−^* mice. Moreover, NLRP10 deficiency resulted in higher bacterial survival in DCs *in vitro* and in the lungs *in vivo* compared to WT and was accompanied by enhanced infiltration of neutrophils and monocytes in the lungs.

Although the initial recruitment of neutrophils and inflammatory monocytes during infection provides the first line of defense against *Mtb* infection (39), this recruitment is also associated with enhanced mycobacterial load and disease exacerbation (21, 40). In addition, IFNγ directly inhibits pathogenic neutrophil accumulation in the infected lung and impairs neutrophil survival (41). Thus, our data suggest that low IFNγ production in *Nlrp10^−/−^* mice could be responsible for the enhanced infiltration of neutrophils and myeloid cells seen in our experiments (42). Likewise, a significant increase in infiltrating neutrophils was also evident in lesioned ears of *Nlrp10^−/−^* mice during the cutaneous inflammatory response induced by *L. major* infection (13), indicating that NLRP10 may regulate a general innate immune response against intracellular pathogens.

Dendritic cells produce IL-12p70 following exposure to PAMPs. No previous physiological functions in inflammation or innate immune defenses have been established for NLRP10. Here, we elucidated NLRP10 as a specific positive regulator of NF-κB activation in DCs upon stimulation of TLR9 by CpG DNA, thereby supporting optimal IL-12p70 and other cytokines. We found that requirement of NLRP10 for IL-12p40 and IL-12p35 synthesis *in vitro* varied depending on the stimulus used. NLRP10 is required for IL-12p40 release from CD40-stimulated DCs and T-cell-mediated stimulation, but it is dispensable to CpG-stimulated and LPS-stimulated DCs. Moreover, IL-12p40 production by PolyI:C-stimulated DCs was also dependent on NLRP10; CpG-induced IL-12p35 synthesis by DCs involves NLRP10. Interestingly, production of the IL-12p40/IL-12p35 heterodimer was undetectable in DC cultures upon CD40 stimulation, suggesting that the active IL-12p70 cytokine requires a microbial priming signal, as previously described (28). We found that *in vivo*, NLRP10 is required by DCs for efficient release of IL-12p40 and IL-12p35 following *Mtb* infection and CpG, but not LPS, administration. The differential requirement of NLRP10 in DCs for IL-12 production could be explained by the fact that it may be required for intracellular TLR-mediated signaling rather than as a trigger by extracellular TLRs. Another possible explanation is that the downstream signaling of TLR4 is dependent on both MyD88 and TRIF (1), which promotes NF-κB and IRF activation, respectively (43). By contrast, TLR9 interacts with MyD88 (its only adaptor molecule) to activate NF-κB signaling, whereas TLR3 engagement with PolyI:C induces the TRIF pathway. Thus, it is possible that the engagement of both the MyD88 and TRIF pathways in LPS-stimulated DCs may bypass the requirement of NLRP10 for NF-κB activation. Indeed, human and mouse DCs produce maximal levels of IL-12p70 when IL-12p40 and IL-12p35 are induced by ligands that engage both MyD88 and TRIF signaling (44). Moreover, the differential requirement of NLRP10 for cytokine production may be explained by considering the differential ability of certain stimuli to engage different signaling adaptors that lead to the canonical and/or non-canonical NF-κB pathways. A natural direction for future investigations will be to ascertain the role of NLRP10 in the activation of the NF-κB pathway primarily triggered by microbial signals.

Other NLRs besides NLRP10 have been implicated in NF-κB signaling. Stimulation of NOD1 and NOD2 by peptidoglycan-derived molecules (iE-DAP dipeptide and muramyl dipeptide, respectively) results in the activation of NF-κB and p38 MAPK *via* the receptor-interacting kinase RIP2, which in turn leads to NF-κB-mediated transcription of numerous pro-inflammatory genes (2). One study showed that ectopically expressed human NLRP10 colocalizes with NOD1 in HeLa cells upon infection with *Shigella flexneri* (12). Oligomerization of NACHT and PYD domains and ATPase activity of NLRP10 are required to stabilize this interaction (12). NLRP10 can also interact with other components of the NOD1 signaling cascade, including RIP2, TAK1, and the NF-κB activating protein NEMO/IKKγ (12). Abrogation of NLRP10 expression in epithelial cells reduced IL-8 and IL-6 release due to diminished activation of p38 MAPK, as well as NF-κB–RelA/p65 activity upon HeLa cell infection with *S. flexneri* (12). As these studies have been conducted using non-immune cell lines, new approaches that are designed to analyze the contribution of NLRP10 in the signaling pathways involved in the innate and adaptive immune responses are warranted.

In conclusion, our study has identified an unprecedented and biologically relevant *in vivo* role for NLRP10 as a regulator of IL-12 release by DCs to promote IFNγ production by Th1 cells. Improved understanding of the immune mechanisms regulated by this NLR that is still largely uncharacterized will advance our current knowledge in pathogen recognition and regulation of innate and adaptive immune responses.

## Ethics Statement

All experimental procedures were approved by the IBC and IACUC of the BRC (A*STAR) in compliance with their Guidelines for Animal Experiments.

## Author Contributions

Conceptualization: GDL, AS, and AM. Methodology: MV, HK, AH, AS, and AM. Investigation: MV, JB, LZ, HK, BP, FL, AH, KN, KL, MM, and LT. Formal analysis: BL and MP. Resources: LS. Data curation: FZ. Writing—original draft: AM. Writing—review and editing: MV, HK, GDL, and AS. Funding acquisition: GDL, AS, and AM. Supervision: AS and AM.

## Conflict of Interest Statement

The authors declare that the research was conducted in the absence of any commercial or financial relationships that could be construed as a potential conflict of interest. The reviewer AQ and handling editor declared their shared affiliation.
