# Supplementary material for: NLRP10 Enhances CD4+ T-Cell-Mediated IFNγ Response via Regulation of Dendritic Cell-Derived IL-12 Release
Source: Front Immunol. 2017 Nov 2;8:1462. doi: 10.3389/fimmu.2017.01462 (PMC5673625; doi:10.3389/fimmu.2017.01462)
Supplement: Supplementary file 1 [file data_sheet_1.pdf]

SUPPLEMENTARY MATERIAL

NLRP10 enhances CD4$^+$ T cell-mediated IFNγ response via regulation of dendritic cell-derived IL-12 release

Maurizio Vacca, Julia Böhme, Lia Paola Zambetti, Hanif Javanmard Khameneh, Bhairav Paleja, Federica Laudisi, Adrian WS Ho, Kurt Neo, Keith Weng Kit Leong, Mardiana Marzuki, Bernett Lee, Michael Poidinger, Laura Santambrogio, Liana Tsenova, Francesca Zolezzi, Gennaro De Libero, Amit Singhal, Alessandra Mortellaro.

SUPPLEMENTARY FIGURES

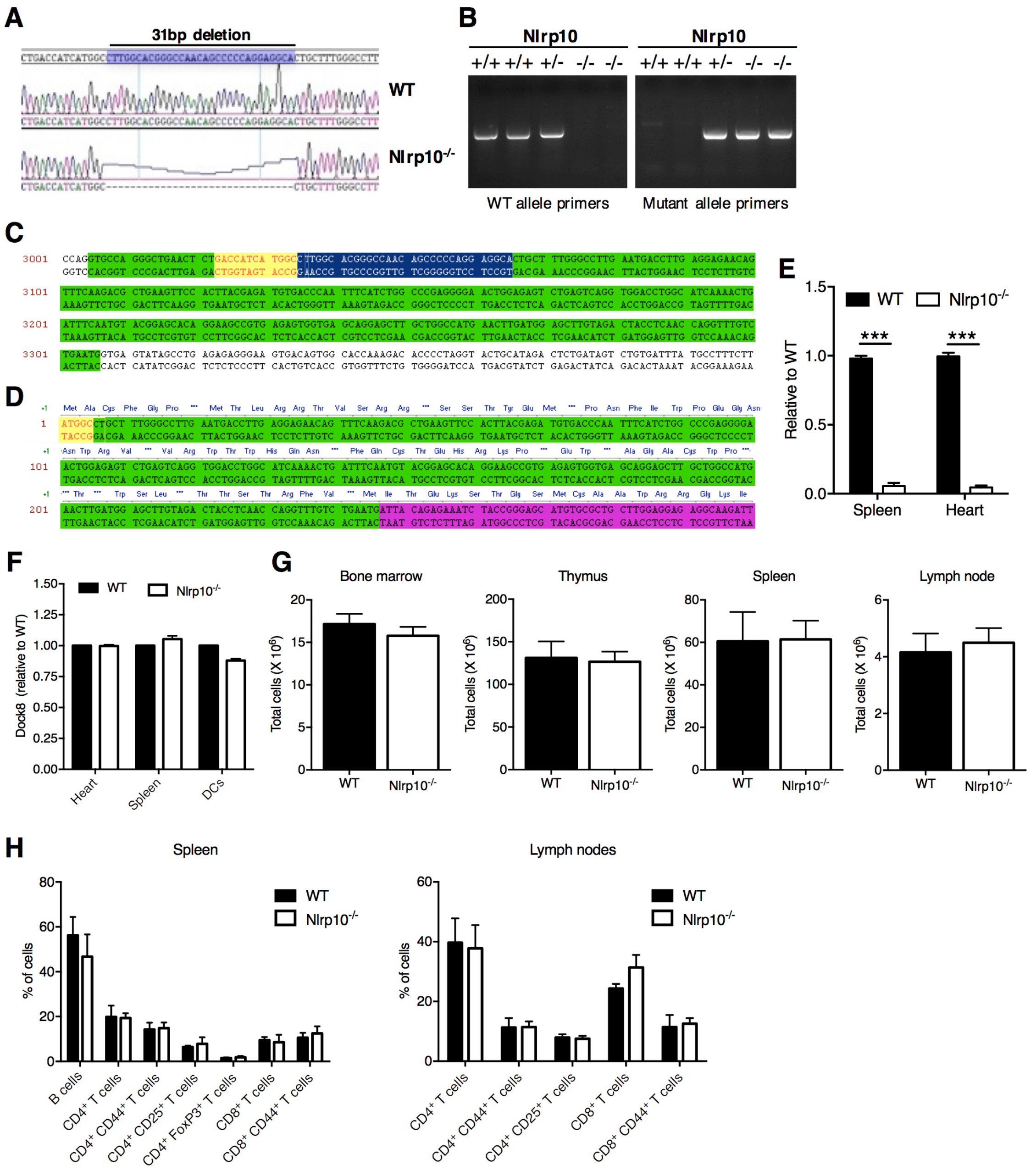

Figure S1. Characterization of *Nlrp10*$^{-/-}$ mice. (A, B) Sequencing (A) and PCR (B) results showing 31 bp deletion in the exon 2 sequence of *Nlrp10* in *Nlrp10*$^{-/-}$ mice. (C) WT genomic sequence of murine *Nlrp10* exon 2 (green). The blue plus yellow highlighted region represents the ZNF target site, and the text highlighted in blue only represents the 31 bp deletion mutation at 3035-3065 bp in DNA. (D) *Nlrp10* mRNA sequence with deletion mutation. The magenta highlighted region indicates the start of exon 3. The translated amino-acid sequence after deletion is shown above the mRNA sequence. Early STOP codons are indicated by *** in exon 2. (E) *Nlrp10* expression was assessed in total splenocytes and heart of *Nlrp10*$^{-/-}$ mice and compared with WT mice by quantitative real-time RT-PCR. (F) mRNA expression of *Dock8* in heart, spleen and bone marrow DCs from WT and *Nlrp10*$^{-/-}$ mice. (G) The cellularity of the indicated primary and secondary lymphoid organs from *Nlrp10*$^{-/-}$ mice was compared with gender and age-matched WT controls. (H) Immunophenotypic characterization of lymphocytes in the spleen and lymph nodes of WT and *Nlrp10*$^{-/-}$ mice. Percentage of B cells, CD4^{+} and CD8^{+} T cells expressing or not CD44 or CD25, and FoxP3 regulatory T cells is shown. Data represents the means ± SD; n=5/group; ***, $p<0.001$. Abbreviations: DC, dendritic cell; WT, wild type.

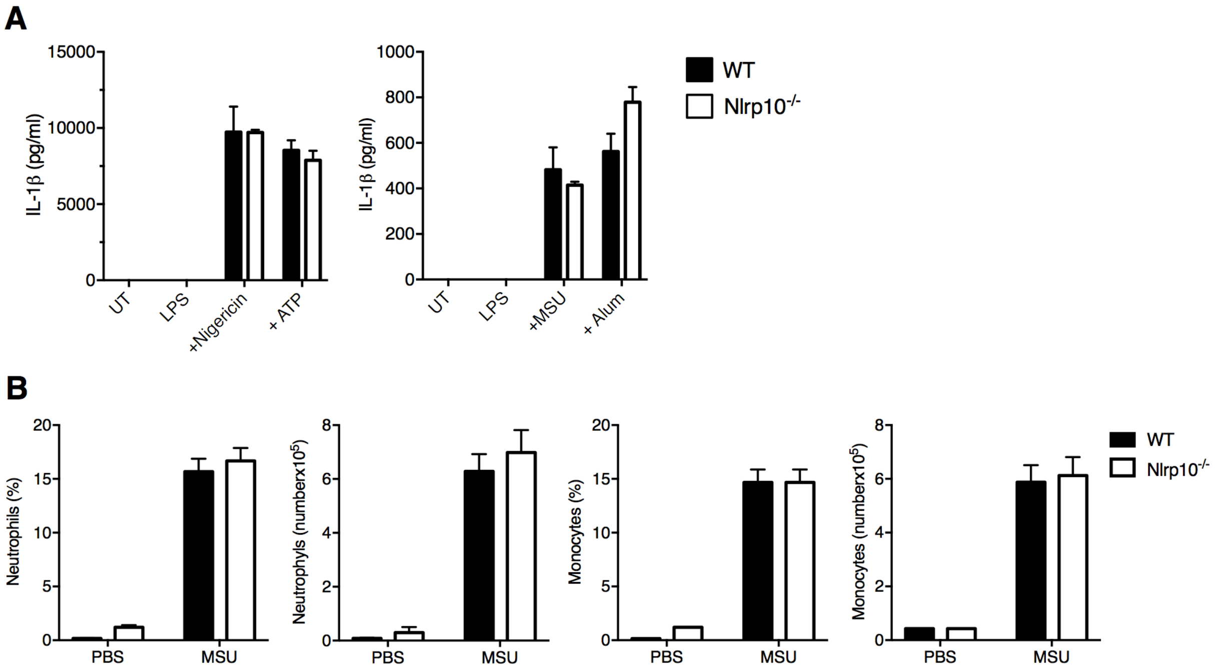

Figure S2. Activation of NLRP3 and IL-1β production is normal in *Nlrp10*$^{-/-}$ mice. (A) Bone marrow-derived dendritic cells were primed or not with LPS (0.1 µg/ml) for 4 h and stimulated with nigericin (10 µM) for 1 h, ATP (5 mM) for 30 min, or MSU crystals (150 µg/ml) or alum (200 µg/ml) overnight. IL-1β levels were assessed in cell-free supernatants by ELISA. (B) MSU crystals (3 mg) were injected i.p. into WT or *Nlrp10*$^{-/-}$ mice. After 6 h, peritoneal cells were collected by lavage, and the numbers of neutrophils ($CD11b^+Ly6G^+$) and monocytes ($CD11b^+Ly6C^+Ly6G^-$) were counted by flow cytometry. The mean total number of cells for each condition is also shown as means ± standard error (n = 4 mice per condition). Abbreviations: Alum, aluminum hydroxide adjuvant; LPS, lipopolysaccharide; MSU, monosodium urate; UT, untreated; WT, wild type.

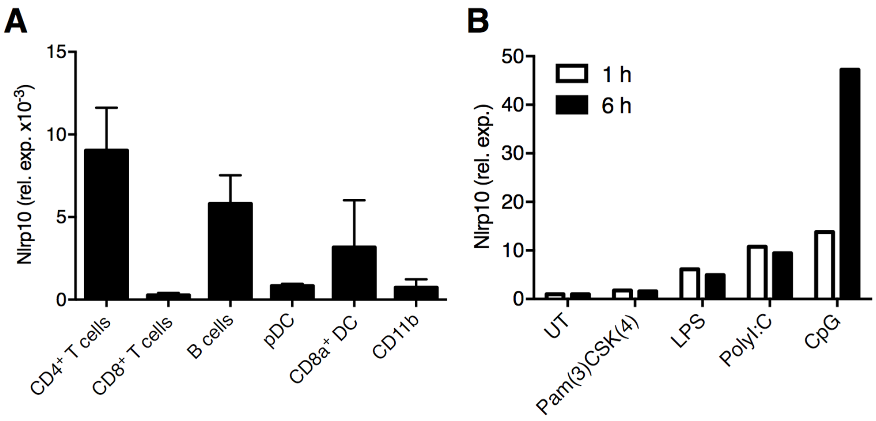

Figure S3. ***Nlrp10*** **expression in mouse immune cells.** Relative expression (rel. exp.) of *Nlrp10* in various immune-cell subsets isolated from spleen (A), and bone marrow-derived DCs stimulated with various Toll-like-receptor agonists for 1 and 6 h, as assessed by quantitative real-time RT-PCR (B). The relative expression levels were evaluated using the $2^{-\Delta Ct}$ (A) or the $2^{-\Delta\Delta Ct}$ (B) methods in which values are normalized to the housekeeping gene *Gapdh* or the unstimulated control, respectively. Abbreviations: DC, dendritic cell; LPS, Lipopolysaccharide; pDC, plasmacytoid DC; UT, untreated.

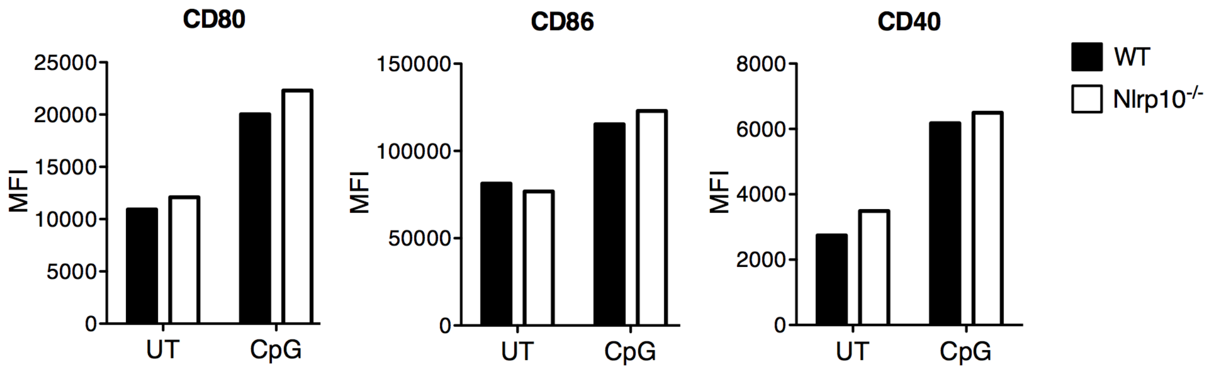

Figure S4. Expression of co-stimulatory markers in Nlrp10$^{-/-}$ and WT dendritic cells. Bone marrow-derived dendritic cells were left untreated or stimulated with CpG (5 µM) for 12 hours and CD80, CD86 and CD40 expression was assessed by flow cytometry. Abbreviations: MFI, mean fluorescence intensity; UT, untreated; WT, wild type.

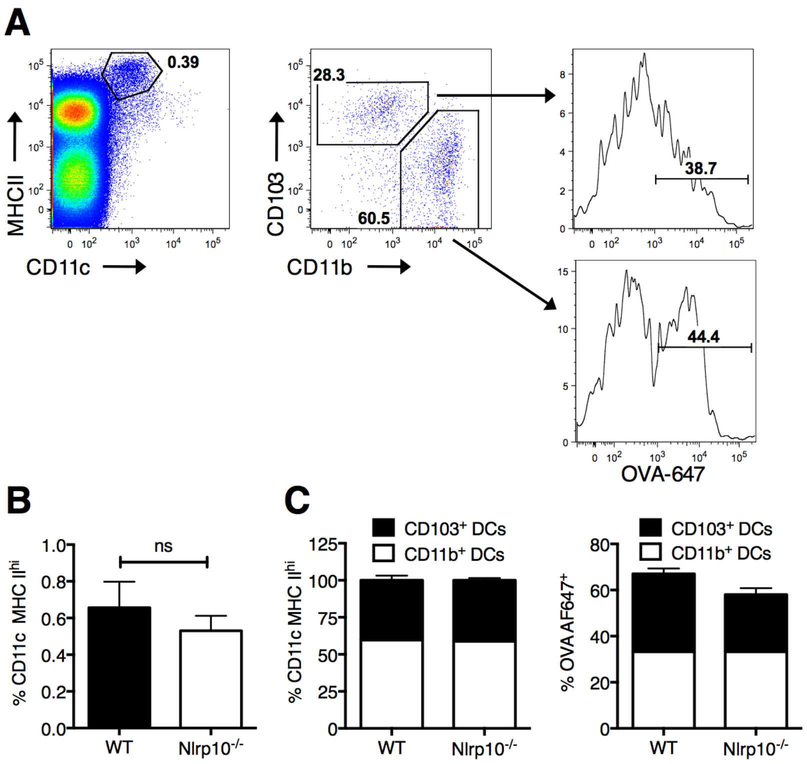

Figure S5. Pulmonary DCs of *Nlrp10*$^{-/-}$ mice can migrate normally *in vivo*. WT and *Nlrp10*$^{-/-}$ mice were treated intratracheally with *E. coli* lipopolysaccharide (10 μg/mouse) and OVA-AF647 (10 μg/mouse). After 24 h, mice were euthanized and the mediastinal lymph nodes were harvested. Lymph nodes were passed through a 70 μm cell strainer to produce a cell suspension. The cells were labeled with anti-CD11c, anti-MHCII, anti-CD11b and anti-CD103 antibodies and analyzed by flow cytometry. (A) Gating strategy. The proportion of total (B) and migratory AF647^{+} (C) cells was determined in CD11c^{high}MHCII^{+}CD103^{+} and CD11c^{high}MHCII^{+}CD11b^{+} DC subsets. Data represent the means ± SD. Abbreviations: DCs, dendritic cells; ns, not significant; OVA, ovalbumin; WT, wild type.

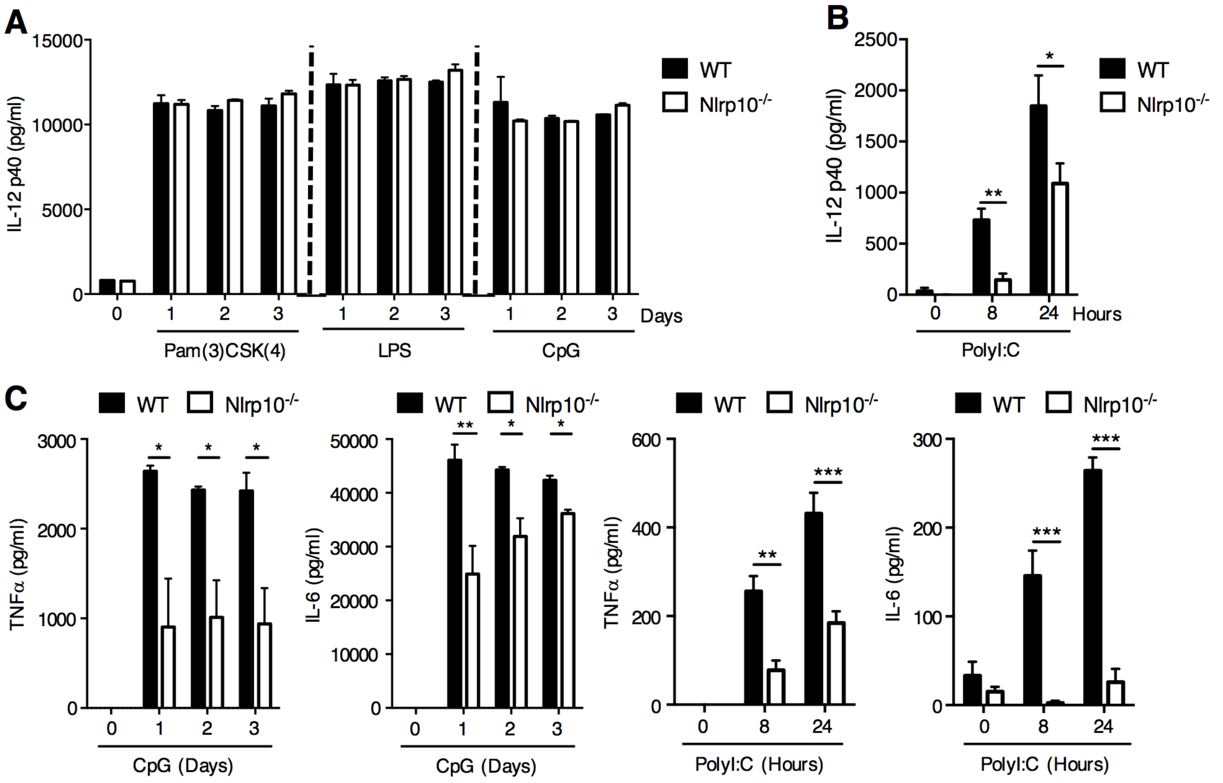

Figure S6. Cytokine production by WT and *Nlrp10$^{-/-}$* dendritic cells stimulated with Toll-like receptor agonists. (A-C) WT and *Nlrp10$^{-/-}$* DCs were stimulated with Pam(3)CSK(4) (1 μg/ml), LPS (100 ng/ml), CpG (5 μg/ml) or PolyI:C (50 μg/ml) for the indicated times. IL-12p40 (A, B), TNFα and IL-6 (C) release was assessed in culture supernatants by ELISA. Data represent the means ± standard deviation. *, $p<0.05$; **, $p<0.01$; ***, $p<0.001$. Abbreviations: LPS, lipopolysaccharide; WT, wild type.

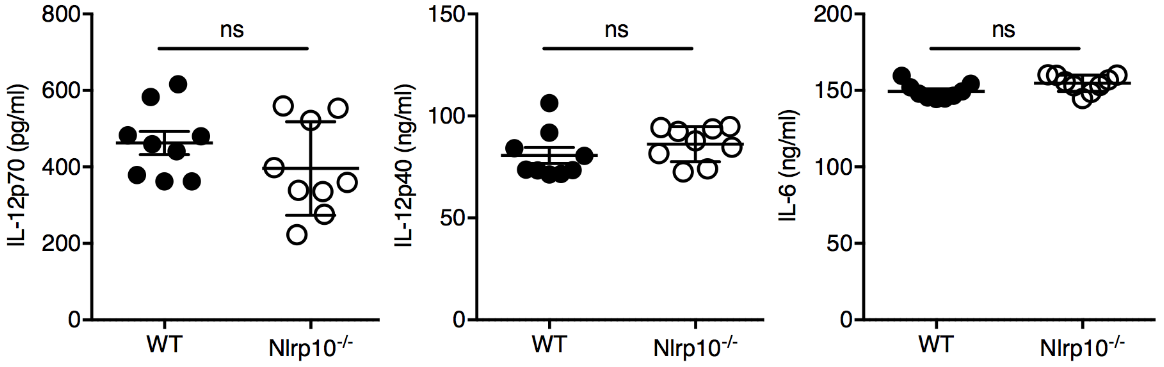

Figure S7. NLRP10 is dispensable for LPS-induced cytokine production *in vivo*. WT and *Nlrp10*$^{-/-}$ mice were injected i.p. with LPS from *E. coli* 0111:B4 (0.5 µg/kg) and IL-12p70, IL-12p40 and IL-6 levels were determined in the plasma after 3 h by ELISA. Individual data and means ± SD are shown. Abbreviations: ns, not significant; WT, wild type.

SUPPLEMENTAL MATERIALS AND METHODS

Generation of *Nlrp10*$^{-/-}$ mouse model

Sixteen ZNF pairs targeting *Nlrp10* exon 2 were assembled by PCR and sub-cloned into a pZNF plasmid. All pairs were tested for efficiency of generating double-strand breaks using the Surveyor Mutation assay in cultured mouse Neuro2A cells. The best performing pair sequence (5'GACCATCATGGCCTTGGCACGGGCCAACAGCCCCCAGGAGGCA-3') was microinjected into one-cell stage mouse embryos harvested from C57/BL6J females. Heterozygous mutant mice were screened by PCR and sequencing, and the targeted mutation was found in intron 2 by PCR and sequencing (Figure S1A-S1D in the Supplementary Material). The mutant founder mouse #25 carrying a 31 bp deletion was bred to C57/BL6J mice in order to generate F1 mutants. These animals were intercrossed to generate homozygous knockouts. Congenic wild-type littermate controls (WT) were used in all experiments.

Nlrp10$^{-/-}$ genotyping

Ear clips were collected and incubated overnight in lysis buffer (10 mM Tris, pH8.0, 50 mM KCL, 0.01% Gelatin, 1% Triton X-100, 10% proteinase K), followed by 10 min inactivation with proteinase K at 95°C. Mice were genotyped by PCR using a common forward primer (F1, 5'-ACCATCATGGCCTTGGCA-3') with a wild-type (WT-R, 5'–GCCACTGTCACTTCCCTCTC-3') or knockout (KO-R, 5'ACCATCATGGC CTGCTTT-3') reverse primer using Promega GoTaq® Hot Start Green Master Mix and the following amplification conditions: 94°C for 2 min and 35 cycles of 94°C for 15 s, 60°C for 15 s, 72°C for 1 min, and 1 cycle at 72°C for 5 min. For sequencing, DNA samples were amplified using a sequencing forward primer (Seq Fwd, 5'-ATCTTGCCTCTCCTCCAAGC-3') and the WT-R primer. PCR products were purified using a Gel/PCR purification kit (Favorgen Biotech. Corp.) and sequenced using the Seq Fwd primer.

Flow cytometry and cell sorting

The following antibody clones from BioLegend, BD Biosciences and eBioscience were used: anti-CD3 (17A2), anti-CD4 (RM4-5 and GK1.5), anti-CD11b (M1/70), anti-CD11c (HL3 and N418), anti-CD40 (3/23), anti-CD45 (30-F11), anti-CD45.1 (A20), anti-CD45.2 (104), anti-CD80 (16-10A1), anti-CD86

(GL1), anti-CD154 (MR1), anti-MHCII I-A/I-E (M5/114.15.2), anti-TCR Vβ5.1/5.2 (MR9-4) and anti-IFNγ (XMG1.2). DAPI was added to the cells 5 min prior to FACS to distinguish live and dead cells.

RNA extraction and semi-quantitative RT-PCR

Total RNA was isolated using Trizol® (Invitrogen) and cleaned using an RNeasy Total RNA Clean-up kit (Qiagen). RNA from heart tissue was isolated using a Qiagen RNeasy Kit. Genomic DNA was removed using a Turbo DNA-free kit (Ambion) before reverse transcription using a High Capacity cDNA Reverse Transcription Kit (Applied Biosystems). qRT-PCR was performed in triplicates using iQ SYBR Green Supermix (BioRad) and the following primers: *Nlrp10* forward 5'-CAACAGCCCCCAGGAG-3', *Nlrp10* reverse 5'-ATCTTGCCTCTCCTCCAAGC-3'; *Il12a* forward 5'-CACGCTACCTCCTCTTTTTGG-3', *Il12a* reverse 5'-GTCTTCAGCAGTGCAGGAATAATG-3'; *Il12b* forward 5'-CAAATTACTCCGGACGGTTC-3', *Il12b* reverse 5'-AGTCCCTTTGGTCCAGTGTG-3'; *Ifng* forward 5'-AGCGGCTGACTGAACTCAGATT-3', *Ifng* reverse 5'-GTCACAGTTTTCAGCTGTATAGGG-3'; *Gapdh* forward 5'-TCGTCCCGTAGACAAAATGG-3', *Gapdh* reverse 5'-TTGAGGTCAATGAAGGGGTC-3'. Amplification was performed on an Applied Biosystems 7500 Real-Time PCR System. The relative expression level of each gene was evaluated by ΔΔCt method, comparing to the *Gapdh* housekeeping gene expression in the untreated condition or the WT control, as indicated.

Confocal microscopy

BMDCs (1.5 x 10^5) were plated in poly-lysine-coated ibidi chambers and treated with CpG DNA (1 μM) for 30 min. Cells were fixed for 10 min in 4% PFA, washed with PBS, and permeabilized in permeabilization buffer (0.1% saponin, 2% BSA in PBS) for 10 min at room temperature (RT). Cells were then incubated with NF-κB p65/RelA antibody (sc-8008, Santa Cruz Biotechnology) diluted 1:200 in dilution buffer (0.01% saponin, 2% BSA in PBS) for 1 h RT. Cells were then washed and incubated for 45 min in the dark with propidium iodide (1 μM) and donkey anti-rabbit FITC-labeled antibody (1:500 in dilution buffer). Finally, the cells were washed and resuspended in 100 μl PBS and kept in the dark at 4˚C. Images were acquired with an Olympus FY1000 system at 40X magnification.

Histological analysis

Formalin-fixed lung tissues of Mtb-infected mice were paraffin-embedded, sectioned and stained with hematoxylin and eosin (H&E) or acid fast staining by Ziehl-Neelsen (ZN) method (IDEXX-RADIL Laboratories Inc, MO) as described previously (Singhal, A. et al. 2014, Sci Transl Med). Stained sections were photographed in a Nikon Microphot-FX photomicrographic system and analyzed with NIS-Elements F3.0 software (Nikon Instruments Inc, NY).

NF-κB Phospho Antibody Array

The cell lysates from WT and *Nlrp10*$^{-/-}$ BMDCs stimulated or not stimulated with CpG DNA (1 μM) for 45 min were carried out a high-throughput proteomic screen of potential *Nlrp10* targets using NF-κB Phospho Antibody Array (FullMoon BioSystems). The Full Moon arrays contain 215 highly specific antibodies against phospho and total proteins involved in the NF-κB pathway. Arrays were prepared following manufacturer's instructions. The raw fluorescence signals were background subtracted and averaged amongst the replicate spots. The averaged background subtracted signals were then logarithmically transformed and then normalized using the house keeping gene β-actin to account for slide to slide variations. Heat maps were generated using the log2 fold changes of the averaged signals and the proteins clustered using hierarchical clustering with Euclidean distance in R 3.3.1.

Western blot

Cytoplasmic cell extracts were prepared using the Cytoplasmic Extraction Buffer (10 mM HEPES-KOH pH 7.9, 60 mM KCl, 1 mM EDTA, 0.5% NP-40) supplemented with 1 mM DTT and protease-inhibitor cocktail. Lysates were prepared by mixing (3 x 2 min) with intermittent incubations on ice (3 min). The cytoplasmic fraction was collected by centrifugation for 10 min at 13,000 rpm at 4°C. The cell pellet was washed with Cytoplasmic Extraction Buffer and resuspended in Nuclear Extraction Buffer (250 mM Tris-HCl pH7.5, 60 mM KCl, 1 mM EDTA) supplemented with 1 mM DTT and protease-inhibitor cocktail. Protein concentrations were determined using a Nanodrop ND1000 (Thermo Scientific). Proteins were boiled in Laemmli buffer containing 5% β-mercaptoethanol for 30 min, separated by SDS–PAGE, and transferred onto PVDF membranes (Bio-Rad Laboratories). Membranes were blocked with 5% non-fat dry milk in PBS with 0.1% Tween 20 (Sigma-Aldrich) for 1 h RT, and incubated overnight at 4 °C with primary antibodies diluted 1:1,000 in 5% BSA: anti-NF-κB p65/RelA (sc-372), anti-NF-κB RelB (sc-226) and anti-lamin B (sc-

6216) from Santa Cruz Biotechnology; anti-IκBα (#4812, Cell Signaling) and anti-GAPDH (MAB374, Millipore). Densitometry was performed using ImageJ and data from cytoplasmic and nuclear extracts were normalized to GAPDH, and lamin B, respectively.

SUPPLEMENTAL TABLE 1. Normalized log2 fluorescence signal. Background subtracted signals were averaged amongst replicate spots and normalized to the house keeping protein β-actin.

**Antibody Name**	**WT UT**	**WT CpG**	**Nlrp10$^{-/-}$ UT**	**Nlrp10$^{-/-}$ CpG**
AKT (Ab-308)	8.38	7.53	8.33	8.14
AKT (Ab-326)	7.68	6.98	7.60	5.33
AKT (Ab-473)	8.40	7.26	8.58	5.18
AKT (Tyr326)	7.65	7.75	8.95	6.69
AKT1 (Ab-124)	8.86	7.63	8.90	5.85
AKT1 (Ab-246)	6.68	5.91	6.63	5.81
AKT1 (Ab-450)	9.13	7.27	8.53	8.04
AKT1 (Ab-474)	5.88	5.65	6.60	3.78
AKT1 (Ab-72)	9.62	8.42	8.90	7.58
AKT1 (Ser124)	8.62	6.15	6.24	7.16
AKT1 (Ser246)	8.32	8.32	8.50	4.83
AKT1 (Thr450)	5.68	7.52	5.43	7.23
AKT1 (Thr72)	9.10	7.30	8.06	-3.24
AKT1 (Tyr474)	6.48	7.91	8.02	-3.24
AKT1S1 (Ab-246)	10.41	10.31	10.71	10.37
AKT1S1 (Thr246)	6.63	7.22	6.92	6.09
AKT2 (Ab-474)	7.52	2.94	7.10	5.17
ATM (Ab-1981)	7.68	5.50	7.30	4.83
BLNK (Ab-96)	7.66	7.93	8.21	8.86
BLNK (Tyr84)	6.92	7.19	6.83	5.33
BLNK (Tyr96)	9.15	7.06	7.85	7.34
BTK (Ab-222)	9.69	8.24	8.87	6.41
BTK (Tyr222)	6.78	7.64	8.10	7.30
BTK (Tyr550)	8.18	7.18	8.17	8.57
Beta actin	12.01	12.01	12.01	12.01
CBP (inter)	8.12	6.48	8.50	4.55
CD40 (C-term)	11.41	10.86	11.50	11.28
CK2-b (Ab-209)	8.00	7.87	7.89	7.59
CK2-b (Ser209)	7.52	6.76	7.21	6.04
COT (Ab-290)	9.47	7.58	9.03	6.32
COT (Thr290)	8.02	6.43	7.07	6.76
Elk-1 (Ab-383)	7.55	5.70	6.61	-3.24
Elk-1 (Ab-389)	8.96	7.50	8.84	4.72
Elk-1 (Ab-417)	8.41	7.08	8.67	5.54
GAPDH	6.12	3.87	5.70	2.66
GSK3 beta (Ab-9)	8.00	5.17	6.30	-3.24
GSK3a-b (Ab-216/279)	7.72	6.92	7.84	6.37
GSK3a-b (Tyr216/279)	8.60	7.52	8.75	4.99
HDAC1 (Ab-421)	10.27	10.67	10.30	11.15
HDAC1 (C-term)	7.49	7.81	7.88	8.34
HDAC1 (Ser421)	9.50	8.41	10.06	6.40

HDAC10 (inter)	7.62	8.25	7.95	8.24
HDAC2 (Ab-394)	8.86	7.76	8.34	7.96
HDAC2 (Ser394)	9.58	8.32	9.10	6.17
HDAC3 (Ab-424)	8.80	8.81	9.16	7.32
HDAC3 (C-term)	8.07	7.17	7.77	7.65
HDAC3 (Ser424)	8.76	7.61	9.11	7.60
HDAC4 (Ab-632)	6.74	5.77	7.25	5.87
HDAC5 (Ab-259)	9.26	7.40	8.58	8.54
HDAC5 (Ab-498)	6.36	6.44	7.04	6.61
HDAC5 (C-term)	10.41	10.83	10.79	11.30
HDAC5 (Ser259)	9.25	7.39	8.61	7.47
HDAC6 (Ab-22)	9.50	9.53	9.91	9.02
HDAC6 (C-term)	5.68	7.51	7.04	7.17
HDAC6 (Ser22)	9.50	7.63	9.35	7.89
HDAC7 (C-term)	10.07	10.33	10.37	10.80
HDAC8 (Ab-39)	7.48	5.80	7.29	5.74
HDAC9 (C-term)	5.18	6.67	7.42	5.72
Histone H3.1 (Ab-10)	5.46	6.03	6.96	6.77
IKK alpha (Ab-23)	6.30	6.37	7.50	6.12
IKK beta (Ab-188)	8.05	6.92	8.07	5.62
IKK beta (Ab-199)	11.25	10.42	10.89	10.75
IKK beta (Tyr188)	8.27	7.72	8.25	7.33
IKK beta (Tyr199)	6.83	7.65	8.15	7.46
IKK gamma (Ab-31)	10.74	11.03	11.19	11.23
IKK gamma (Ab-85)	8.28	7.85	8.54	5.37
IKK gamma (Ser31)	5.86	7.56	7.83	6.87
IKK gamma (Ser85)	5.46	7.45	5.92	7.70
IKK-a/b (Ab-180/181)	8.33	7.59	8.59	6.33
IKK-a/b (-Ser180/181)	5.05	6.81	7.39	6.99
IkB-alpha (Ab-32/36)	5.27	6.57	7.22	7.67
IkB-alpha (Ab-42)	-1.45	6.25	6.69	4.79
IkB-alpha (Tyr305)	8.64	7.04	8.25	7.27
IkB-beta (Ab-19)	8.54	6.23	8.57	5.37
IkB-beta (Ser23)	8.00	5.98	8.32	7.42
IkB-beta (Thr19)	7.55	7.28	6.47	7.20
IkB-epsilon (Ab-22)	11.03	10.14	10.18	10.15
IkB-epsilon (Ser22)	7.93	6.42	7.83	7.97
JNK1/2/3 (Ab-183)	7.79	6.54	7.39	7.12
JNK1/2/3 (Thr183/Tyr185)	6.28	6.62	7.23	5.25
LCK (Ab-192)	7.53	6.39	7.33	5.85
LCK (Ab-393)	9.59	9.36	10.20	9.52
LCK (Ab-504)	7.68	7.05	7.41	6.88
LCK (Ab-59)	7.84	5.72	7.83	5.35
LCK (Ser59)	6.33	6.81	6.48	6.44
LCK (Tyr192)	8.47	7.32	8.43	8.16

LCK (Tyr504)	8.43	7.95	8.69	7.55
MAP3K7/TAK1 (Ab-187)	7.00	6.04	6.68	3.99
MAP3K7/TAK1 (Ab-439)	6.05	6.53	5.34	6.02
MSK1 (Ab-360)	11.09	10.67	11.12	10.91
MSK1 (Ab-376)	5.93	6.83	6.71	-3.24
MSK1 (Ab-581)	7.24	7.63	8.17	7.46
MSK1 (Ser212)	8.22	8.02	8.38	8.79
MSK1 (Ser360)	7.41	8.25	8.82	5.25
MSK1 (Thr581)	9.11	7.45	8.74	5.73
NFkB-p100 (Ser872)	6.63	8.66	8.57	9.36
NFkB-p100/p52 (Ab-865)	10.81	10.09	10.38	9.44
NFkB-p100/p52 (Ab-869)	8.86	7.49	8.70	5.81
NFkB-p105 (Ab-927)	9.09	8.91	9.04	8.23
NFkB-p105 (Ser927)	6.89	7.37	7.58	7.03
NFkB-p105/p50 (Ab-337)	9.71	7.61	9.04	-3.24
NFkB-p105/p50 (Ab-893)	9.55	7.56	9.17	6.34
NFkB-p105/p50 (Ab-907)	9.66	7.58	8.65	-3.24
NFkB-p105/p50 (Ab-932)	9.79	7.75	8.13	4.40
NFkB-p105/p50 (Ser932)	5.96	6.58	6.08	7.36
NFkB-p65 (Ab-254)	9.35	6.64	7.87	4.97
NFkB-p65 (Ab-276)	9.53	6.75	8.00	4.95
NFkB-p65 (Ab-281)	7.58	6.53	7.36	3.24
NFkB-p65 (Ab-311)	9.73	7.38	8.72	2.66
NFkB-p65 (Ab-435)	8.77	7.11	7.80	4.19
NFkB-p65 (Ab-468)	8.88	7.54	8.51	5.88
NFkB-p65 (Ab-505)	8.62	7.97	8.38	7.60
NFkB-p65 (Ab-529)	8.75	7.93	8.23	6.86
NFkB-p65 (Ab-536)	8.55	6.59	8.38	-3.24
NFkB-p65 (Ser311)	8.67	8.78	9.13	9.55
NFkB-p65 (Ser529)	7.67	7.64	7.94	8.32
NFkB-p65 (inter)	8.22	8.48	8.53	9.04
P38 MAPK (Ab-182)	11.52	10.39	11.14	11.89
P38 MAPK (Ab-322)	7.12	6.95	7.67	6.39
P38 MAPK (Thr180)	-1.45	4.40	5.22	4.60
P38 MAPK (Tyr182)	-1.45	6.48	5.94	6.30
P38 MAPK (Tyr322)	9.78	8.02	8.82	-3.24
P38MAPK (Ab-180)	7.41	6.66	6.99	4.60
PI3-kinase p85-alpha (Tyr607)	5.20	7.13	6.12	7.51
PI3-kinase p85-subunit alpha/gamma (Ab-467/199)	8.91	7.28	8.44	6.82
PI3-kinase p85-subunit alpha/gamma (Tyr467/Tyr199)	8.91	6.22	8.53	7.58
PKA CAT (Ab-197)	6.27	7.09	7.06	6.93
PKA CAT (Thr197)	7.47	6.19	6.65	-3.24
PKC alpha/beta II (Ab-638)	6.43	6.47	6.93	4.99
PKC alpha/beta II (Thr638)	6.55	7.40	9.04	-3.24

PKC beta/PKCB (Ab-661)	7.15	5.71	7.56	-3.24
PKC beta/PKCB (Ser661)	-1.45	6.19	6.39	5.49
PKC theta (Ab-538)	11.84	11.61	11.49	11.70
PKC theta (Ab-676)	9.33	6.53	8.54	-3.24
PKC theta (Ser676)	5.07	7.04	7.73	7.01
PKC theta (Thr538)	9.01	7.77	9.28	6.43
PKC zeta (Ab-410)	9.17	7.62	9.10	5.94
PKC zeta (Ab-560)	6.63	6.33	7.25	4.60
PKC zeta (Thr410)	9.10	7.18	8.63	8.26
PKC zeta (Thr560)	8.32	7.29	8.88	5.51
PKR (Ab-446)	10.09	7.12	9.03	4.25
PKR (Ab-451)	9.40	7.33	8.71	4.17
PKR (Thr446)	5.50	5.46	6.70	5.32
PKR (Thr451)	-1.45	7.21	7.25	7.24
PLCG1 (Ab-771)	8.61	6.14	8.15	5.96
PLCG1 (Ab-783)	8.46	6.45	8.31	3.83
PLCG1 (Tyr1253)	5.64	6.57	6.98	6.42
PLCG1 (Tyr771)	-1.45	6.95	6.74	6.51
PLCG1 (Tyr783)	5.58	6.00	6.80	5.63
PLCG2 (Ab-1217)	9.14	7.62	8.81	6.39
PLCG2 (Ab-753)	8.88	6.73	8.64	5.86
PLCG2 (Tyr1217)	7.20	6.61	7.38	6.49
PLCG2 (Tyr753)	9.03	7.14	7.15	8.39
RAS(p21 H and K) (inter)	7.34	6.42	7.43	4.78
RASE (inter)	7.82	6.48	7.60	5.48
Ras-GRF1 (Ab-916)	8.93	7.90	8.45	8.16
Ras-GRF1 (Ser916)	8.88	6.43	8.41	-3.24
Rel (Ab-503)	9.22	7.65	8.49	-3.24
RelB (Ab-552)	8.95	7.58	7.71	-3.24
RelB (Ser552)	6.94	7.18	7.08	7.04
SAPK/JNK (Ab-183)	9.35	7.56	8.31	7.07
SAPK/JNK (Ab-185)	8.80	7.03	7.59	-3.24
SAPK/JNK (Thr183)	6.12	6.29	7.14	7.06
SAPK/JNK (Tyr185)	6.80	4.89	6.85	8.01
STRAD (inter)	9.17	7.21	8.53	8.68
SUMO-1 (N-term)	8.67	7.40	8.32	6.25
SUMO2/3 (Cleaved at G93)	8.61	6.98	8.17	6.99
SYK (Ab-348)	5.27	7.38	8.50	3.78
SYK (Ab-525)	9.33	8.79	9.04	8.56
SYK (Tyr323)	6.64	6.10	6.90	-3.24
SYK (Tyr348)	9.46	7.62	9.29	7.60
SYK (Tyr525)	4.46	7.41	6.39	6.77
SYK (inter)	9.08	8.53	8.64	8.27
TAK1 (Ab-184)	8.52	6.52	7.52	4.39
TAK1 (Thr184)	5.50	6.09	4.86	6.80

TGF alpha (inter)	8.07	8.81	9.11	7.82
TGFBR1 (Ab-165)	8.52	7.33	8.05	5.94
TGFBR2 (Ab-250)	9.59	9.27	9.54	9.48
TNF Receptor II (C-term)	10.01	9.55	9.94	9.89
TNF receptor-1 (inter)	7.93	5.70	7.48	6.79
TRADD (inter)	7.86	6.98	8.27	6.66
Ubiquitin (inter)	8.16	7.47	7.65	6.64
ZAP-70 (Ab-292)	4.88	7.04	7.87	6.93
ZAP-70 (Tyr292)	8.14	7.60	8.22	-3.24
ZAP-70 (Tyr315)	8.13	7.32	8.13	4.66
Zap-70 (Ab-319)	7.64	7.81	8.31	7.54
Zap-70 (Ab-493)	6.30	6.58	7.36	-3.24
p300/CBP (C-term)	7.30	5.66	7.13	2.08
